# Electrochemical Sensor Based on a Composite of Babassu Starch, Carbon Black, and Cobalt Phthalocyanine for the Detection of Hydroxychloroquine

**DOI:** 10.1021/acsomega.5c13354

**Published:** 2026-02-12

**Authors:** João Pedro C. Silva, Gilvana P. Siqueira, Domingos Rosa Santos-Neto, Raquel G. Rocha, Jéssica S. Stefano, Luiza M. F. Dantas, Luiz Ricardo G. Silva, Eduardo M. Richter, Rodrigo A. A. Muñoz, Iranaldo S. da Silva

**Affiliations:** † Department of Chemical Technology, Federal University of Maranhão, São Luís, Maranhão 65080-805, Brazil; ‡ Institute of Chemistry, Federal University of Uberlândia, Uberlândia, Minas Gerais 38408-100, Brazil

## Abstract

This work presents the development of an electrochemical sensor based on a starch film extracted from the Babassu coconut mesocarp flour, carbon black (CB), and cobalt phthalocyanine. Scanning-electron microscopy coupled with energy-dispersive spectroscopy confirmed the presence of each component in composite, by confirming alteration of surface morphology and composition. Raman and Fourier-transform infrared spectroscopy also evidenced surface alteration while atomic-force microscopy revealed increase in rugosity after insertion of CB and cobalt phthalocyanine. The developed sensor was applied for the determination of hydroxychloroquine (HCQ) in both environmental and pharmaceutical (tablet) samples, using linear sweep voltammetry and a 0.1 mol L^–1^ phosphate buffer solution (pH = 7.0) as the supporting electrolyte. The method achieved impressive detectability in comparison with literature, with limits of detection (LOD) and quantification (LOQ) of 0.015 μmol L^–1^ and 0.052 μmol L^–1^, respectively. Recovery studies conducted in pharmaceutical matrices, tap water, and treated water samples yielded values ranging from 100% to 105%, demonstrating the sensor’s accuracy. Notably, no electrode fouling was observed after HCQ oxidation, confirming the stability of the modified electrode. These findings underscore the potential of this sensor for environmental monitoring and highlight starch as a sustainable alternative material for immobilizing modifiers on electrodes.

## Graphical abstract

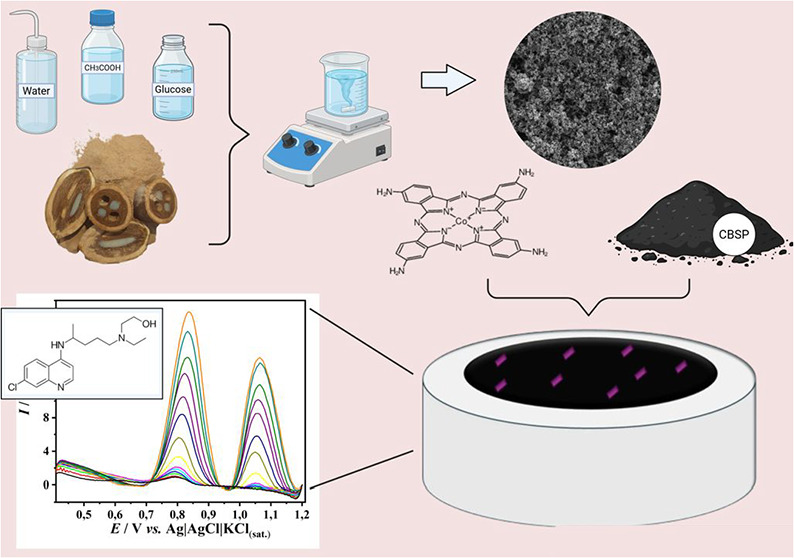

## 1 Introduction

Film-based modifications to support nanomaterials on electrode surfaces have been widely applied in the development of advanced electroanalytical devices.^1–3^ Numerous polymeric materials, including Nafion,^4,5^ dihexadecyl phosphate^6^ and poly­(allylamine) hydrochloride^7,8^ in combination with conductive materials (carbon nanotubes, CB, graphene oxide, reduced graphene oxide, etc.) have been employed for this purpose. Thin films composed of biomaterials have recently attracted significant of interest as sustainable alternatives to reduce reliance on hazardous reagents and mitigate environmental impact. Starch, the second most abundant biopolymer in the world after cellulose, plays a key role in this context. It is a nontoxic, natural polysaccharide easily extracted from seed (cereal grains), stem-pith, fruits, tuber and root.^9^ This material is generally composed of amylose, a linear polymer formed by glucose residues and amylopectin.^10^ Both polymers contribute to the absorption capacity, water solubility and mechanical properties of the starch films. Starch obtained from potatoes,^11,12^ cassava^13^ and/or maize^14^ have already been explored to use as electrode modifications.

Babassu (*Orbignya sp*.) is an important native palm tree from Amazon region and has a great potential for use in industrial applications, including biodiesel production.^15^ For around 300,000 people in the State of Maranhão, Brazil,^16^ engaging with babassu is not only a vital economic activity but also a significant social and political act that supports and complements subsistence farming. Moreover, industry projections indicate strong growth in the babassu oil market, with its value expected to rise from 227.7 million in 2022 to 347.0 million by 2032.^16,17^ Babassu fruit contains a mixture comprising approximately 60% starch.^18^ Despite its traditional regional use, the properties of babassu starch remain largely unexplored for the development of modified electrodes.^19^ To date, only one reported application exists: Lima et al.^19^ demonstrated the successful modification of an indium tin oxide (ITO) electrode with a babassu starch film for the electrochemical detection of the antineoplastic drug methotrexate.

Nanomaterials are widely used in the construction of modern and efficient electrochemical devices due to their unique properties,^20^ such as enhanced surface area, greater intrinsic mobility, and improved electrical conductivity. Modified electrodes based on carbon nanomaterials, such as carbon nanotube fibers and functionalized graphene oxides, have been widely investigated due to their high surface area, excellent electrical conductivity, and versatility in surface modification.^21–24^ These composites offer several advantages^25,26^ and can also promote catalytic effects in certain analytes redox reactions.^27^ CB is a low-cost nanostructured material with excellent electrochemical properties. It is commonly used by various research groups to modify electrodes with carbon paste materials.^28–31^ CB is primarily derived from combustion of petroleum products and consists of spherical aggregates of ten or more carbon particles, with diameters ranging from 3 to 100 nm.^32^ It is predominantly composed of sp^2^-hybridized carbon atoms, with a smaller fraction of sp^3^-hybridized atoms. Moreover, its surface area can vary from 15 to 1000 m^2^ g^–1^, and its electrical conductivity (1.0 × 10^3^ S m^–1^) is higher than that of carbon nanotubes (0.25 S m^–1^).^33^

Cobalt phthalocyanine (Co-Pc) is an attractive catalyst material, often employed in the electrochemical oxidation of a variety of analytes,^34–37^ especially those with extended π-systems that allow for quick redox reactions with little reorganization energy.^38^ Nevertheless, Co-Pc has poor conductivity and a tendency to aggregate in real-world applications. To overcome these problems, Co-Pc is frequently bonded to different carbon surfaces such as graphite, graphene, CB, and carbon nanotubes.^38^

The polymerization of cobalt phthalocyanine into a sheet-like poly-CoPc structure has been shown to significantly enhance its electrochemical stability and redox activity, preserving the intrinsic electrocatalytic properties of the phthalocyanine macrocycle. When immobilized onto conductive substrates, such polymeric cobalt phthalocyanines promote efficient charge transfer and provide a robust electroactive interface, reinforcing the versatility of cobalt phthalocyanine materials for electrocatalytic and sensing applications.^39–41^

Hydroxychloroquine (HCQ) is a bactericidal, antiseptic, and antipyretic drug that is predominantly used to treat malaria, which is endemic in Latin America, with Brazil accounting for 20% of reported cases.^42^ During the COVID-19 pandemic, in vitro experiments indicated that HCQ may be effective against the SARS-CoV-2 virus.^43^ As a result, it became part of early treatment procedures, and its use grew dramatically throughout the pandemic. However, pharmacokinetic investigations have revealed that the HCQ dosage used to treat the SARS-CoV-2 virus may pose risks to human health, such as cardiovascular complications, retina damage, and respiratory disorders.^44^

The presence and distribution of HCQ and its metabolites in aquatic ecosystems have increased, largely due to the discharge of hospital and domestic waste, as well as sewage, into rivers and streams. In many regions, these pollutants are inadequately treated,^45,46^ and the technologies required to remove them are often either too expensive or insufficiently efficient.^47^ As a result, HCQ and its metabolites are classified as emerging contaminants. Nason et al. used liquid chromatography to identify HCQ concentrations of up to 50 μg L^–1^ in environmental samples during the COVID-19 pandemic in Connecticut, USA.^48^ Currently, HCQ detection methods include liquid-phase microextraction coupled with high-performance liquid chromatography (HPLC) and fluorescence detection.^49^ Recently, there has been a surge of interest in the development of analytical sensors for real-time monitoring of diverse analytes, such as those related to water quality, food safety, forensics, and medicines.^50^ Electrochemical methods have several key properties that are important for on-site measurements, including minimal sample manipulation, high sensitivity, low cost, and easy miniaturization. These techniques are based on the detection of electrical signal changes resulting from interactions between analytes and electrodes.^51^

Herein, we report on the fabrication and electrochemical characterization of a novel electrode composite that integrates Babassu starch as a sustainable biopolymer matrix for the immobilization of CB Super P and cobalt phthalocyanine onto a glassy carbon electrode (GCE) surface. As proof of concept, the resulting electrochemical sensor demonstrated excellent performance for the detection of HCQ in environmental samples, highlighting its potential for practical applications in real-world monitoring.

## 2 Experimental Section

### 2.1 Chemicals and Samples

All aqueous stock solutions were prepared using high-purity deionized water (resistivity ≤ 18.2 MΩ cm) from a Millipore Direct-Q3 water purification system (Bedford, MA, USA). Hydroxychloroquine sulfate (100 wt %) was obtained from Sigma-Aldrich (St. Louis, MO, USA). Potassium chloride (99.5 wt %) was acquired from Carlo Erba (Emmendingen, Germany). Potassium ferrocyanide (99 wt %), phosphoric acid (85% w/v), and acetic acid (99.8% w/v) were obtained from Labsynth (São Paulo, Brazil). Sodium dihydrogen phosphate monohydrate (98–102 wt %) and sodium hydroxide (97 wt %) were obtained from ISOFAR (Rio de Janeiro, Brazil). Dimethylformamide (99.8% w/v) was purchased from Êxodo Científico (Sumaré, Brazil). Boric acid was sourced from AppliChem Panreac (Barcelona, Spain), and CB Super P (CBSP) particles were obtained from TIMCAL (Westlake, USA). Babassu mesocarp flour was acquired from a local supermarket (São Luís-MA, Brazil).

The Britton-Robinson (B-R) buffer (2.0 < pH < 12.0) was prepared by mixing acetic, phosphoric, and boric acids, all at 0.04 mol L^–1^; the pH was adjusted with a 1 mol L^–1^ NaOH solution. The 0.10 mol L^–1^ phosphate buffer (PB) pH 7.0 was prepared using sodium dihydrogen phosphate monohydrate, and its pH was also adjusted with a 1.0 mol L^–1^ NaOH solution. The HCQ stock solutions (10.0 mmol L^–1^) were prepared daily by diluting an appropriate amount of HCQ in 0.10 mol L^–1^ phosphate buffer, pH 7.00.

### 2.2 Electrochemical Instrumentation

All electrochemical measurements, including cyclic voltammetry (CV), linear sweep voltammetry (LSV), and electrochemical impedance spectroscopy (EIS), were carried out at room temperature in the presence of dissolved oxygen. Data were collected and processed using an Autolab PGSTAT204 potentiostat/galvanostat (Eco Chemie, Metrohm, Netherlands) controlled by NOVA 2.1.7 software. After baseline correction, all cyclic voltammetry measurements were shown using the “moving average” algorithm and a window size of 2. The reference electrode was a laboratory-made Ag|AgCl electrode in 3 mol L^–1^ KCl, while the auxiliary electrode was a platinum wire.

EIS measurements were performed using an open-circuit potential (0.22 V vs. Ag|AgCl|KCl_(sat.)_) in the presence of redox probe [Fe­(CN)_6_]^3–/4–^ in a 0.1 mol L^–1^ KCl solution, by applying an alternating potential with an amplitude of 10 mV over a frequency range of 50 kHz to 0.1 Hz. The Randles equivalent circuit was used to fit the experimental data and determine the charge transfer resistance (*R*_ct_) related to the [Fe­(CN)_6_]^3–/4–^ species.

### 2.3 Characterizations

Scanning electron microscopy (SEM) images were obtained using a Tescan VEGA 3 LMU operating at 20 kV. Raman spectra were collected using a HORIBA LABRAM HR Evolution Spectrophotometer (Japan), controlled by HORIBA Scientific’s LabSpec software, and equipped with an OSD Syncerity detector using a 532 nm laser operating at 50 mW over the range of 4000 to 80 cm^–1^. Fourier-transform infrared (FTIR) measurements were performed using a PerkinElmer Spectrum Two spectrometer in the attenuated total reflectance (ATR) mode with a CsI detector. Measurements were recorded over the range of 400–4000 cm^–1^ with a resolution of 4 cm^–1^.

Atomic force microscopy (AFM) images were obtained using a Scanning Probe Microscope (SPM-9600, Shimadzu, Japan), in dynamic mode, with silicon PPP-NCHR AFM probes (NANOSENSORS, Switzerland) with a resonance frequency of 330 kHz, a force constant of 42 N/m, a length of 125 μm, an average width of 30 μm, and a thickness of 4 μm.

### 2.4 Preparation of the Modified Electrode

In 15 mL centrifuge tubes, 5 g of babassu coconut mesocarp flour was added, and the tubes were then filled up to 15 mL with distilled water (Figure 1a). The tubes were then placed in a centrifuge at 2000 rpm for 15 min. After this time, the tubes showed a bottom layer, with the upper part having a lighter color indicating the presence of starch (Figure 1b), while the darker lower part contained the insoluble residue from the flour. The solid starch was separated with the help of a spatula.

**Figure 1:**
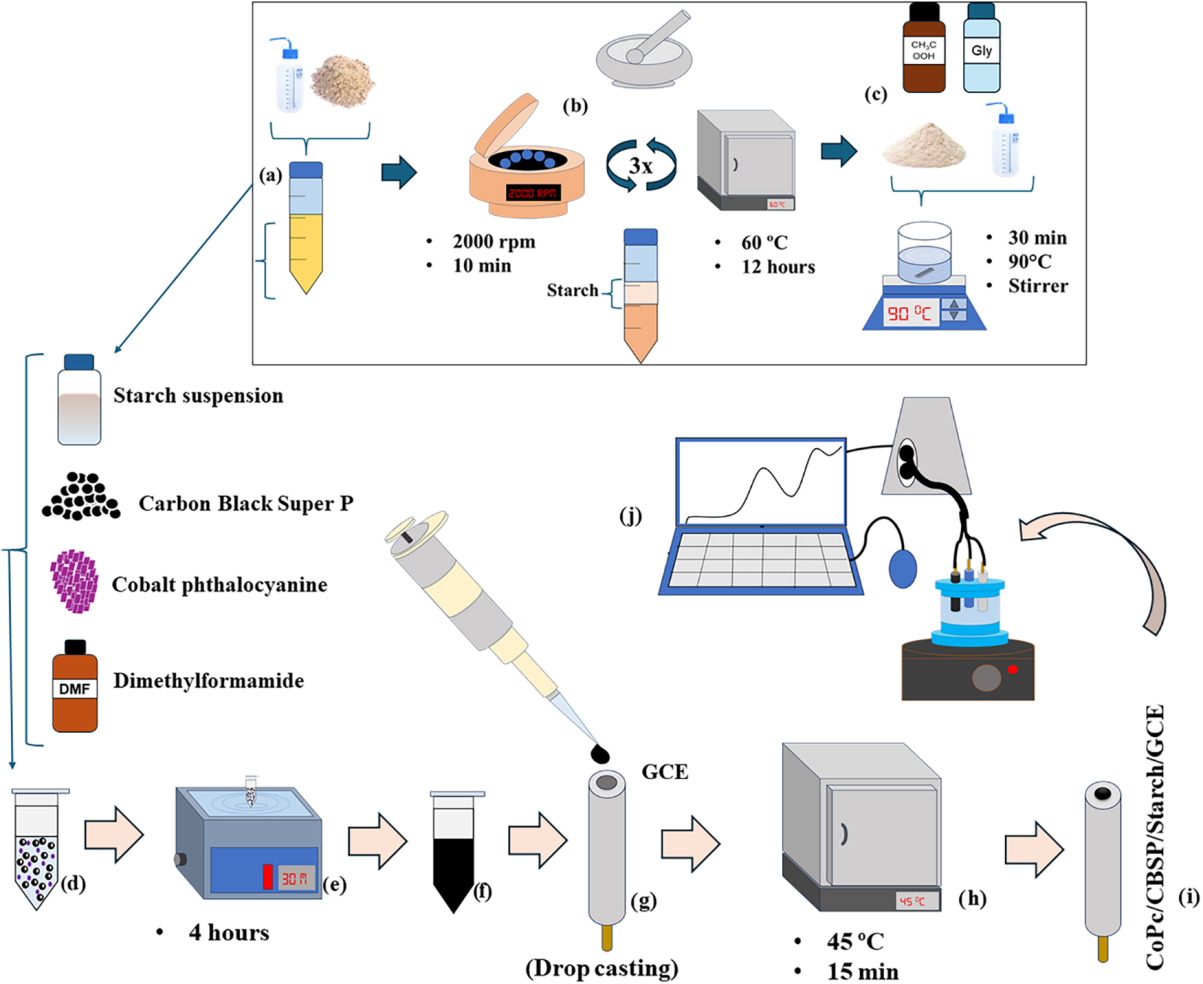
Experimental procedure: (a–c) starch suspension preparation; (d–f) preparation of the modifying suspension; (g–i) schematic representation of the modified electrode preparation; (j) electrochemical measurement.

The extracted starch was dried in an oven for 12 h at 60 °C. After that, it was ground using a mortar and pestle. The dried starch underwent two additional washing and centrifugation steps to obtain purer starch material.

After the starch was extracted, a suspension was made by adding 50 mg of starch, 1 mL of glycerol, 5 mL of acetic acid, and the volume was completed with 94 mL of distilled water into a beaker (Figure 1c). The mixture was heated at 90 °C for 30 min under constant stirring until a homogeneous suspension was obtained. Initially, a portion of the starch suspension was diluted to 200 mg L^–1^.

To prepare the modifier material, 5 mg of CBSP was added to a 2 mL centrifuge tube, along with 1 mg of cobalt phthalocyanine (CoPc), 250 μL of a starch suspension at a concentration of 200 mg L^–1^, and the mixture was completed with 750 μL of dimethylformamide (DMF) (Figure 1d). The mixture was then sonicated for 4 h (Figure 1e) in an ultrasonic bath until the suspension was completely homogenized (Figure 1f).

To modify the GCE, a 5 μL aliquot of the suspension was added to the electrode’s surface using the drop-casting approach (Figure 1g). The electrode was then dried in an oven at 45 °C for 15 min (Figure 1h,i). Once dry, the electrode was ready for use as a sensor in a three-electrode electrochemical cell (Figure 1j). For electrodes that were not prepared immediately after the first suspension preparation, an additional homogenization procedure was performed using a vortex shaker.

For the preconcentration time (*T*_PC_) step, the CoPc/CBSP/Starch/GCE electrode was stirred in the electrochemical cell in the presence of the HCQ solution, while a potential of +0.39 V was applied for a predefined time of 160 s. Afterward, voltammetric measurements were performed.

### 2.5 Sample Preparation

HCQ tablets were purchased from a local drugstore (Maranhão, Brazil). Ten tablets were weighed and ground. Based on the weight and the labeled information, a 10 mmol L^–1^ HCQ stock solution was prepared in 2 mL solution. After that, the stock solution was diluted in the electrochemical cell and the HCQ sample concentration was determined using the standard-addition calibration method.

A tap water sample was collected from the Institute of Chemistry at the Federal University of Uberlândia (Uberlândia, Brazil). The collected water was used to prepare a 0.1 mol L^–1^ phosphate (PB) buffer at pH 7.0, which was then spiked with known concentrations of HCQ (0.2 and 1.0 μmol L^–1^).

A water sample from the treatment plant was provided by the Department of Water and Sewage of Uberlândia (Uberlândia, Brazil) and stored in a refrigerator. It was used to prepare a 0.1 mol L^–1^ PB buffer at pH 7.0, which was then spiked with 0.1 and 0.2 μmol L^–1^ HCQ.

## 3 Results and Discussion

### 3.1 Characterization

The morphological properties of the materials were investigated using SEM images (Figure 2) at various magnifications. Figure 2A shows starch granules extracted from the mesocarp of the babassu coconut, with an average size of approximately 10 μm. Their morphology is consistent with patterns reported in the literature.^52^ Figure 2B illustrates the surface of the starch film, revealing an apparently porous and irregular surface morphology, with a larger area compared to the clean GCE. In this condition, the starch granules undergo gelatinization, forming a continuous film that contributes to the observed morphological changes.^53^ The presence of grooves is evident, and the physical structure is consistent with that reported in the literature. Upon closer inspection at higher magnification (Figure 2C), the clustered particles that form the CBSP become more apparent, which is in agreement with findings reported in the literature.^54^ Figure 2D shows the fully modified electrode (CoPc/CBSP/Starch/GCE), where one can see a similar structure to that shown in Figure 2C, but with the presence of crystalline segments in a baston form. This result reveals the incorporation of cobalt phthalocyanine (CoPc) into the modifier, which is consistent with previous literature reports. The CoPc crystals are uniformly distributed and adsorbed onto the CBSP/Starch composite, highlighted in red, as shown in Figure 3D. AFM results indicate that the modified electrode exhibits a significantly higher electroactive area compared to the bare GCE, due to increased surface roughness and morphological features introduced by the modification, despite both electrodes having the same geometric area. The dominant elements at each stage can be observed by elemental analysis performed via Energy Dispersive Spectroscopy (Figure S1). A discussion of the electrode composition is provided in the Supporting Information.

**Figure 2:**
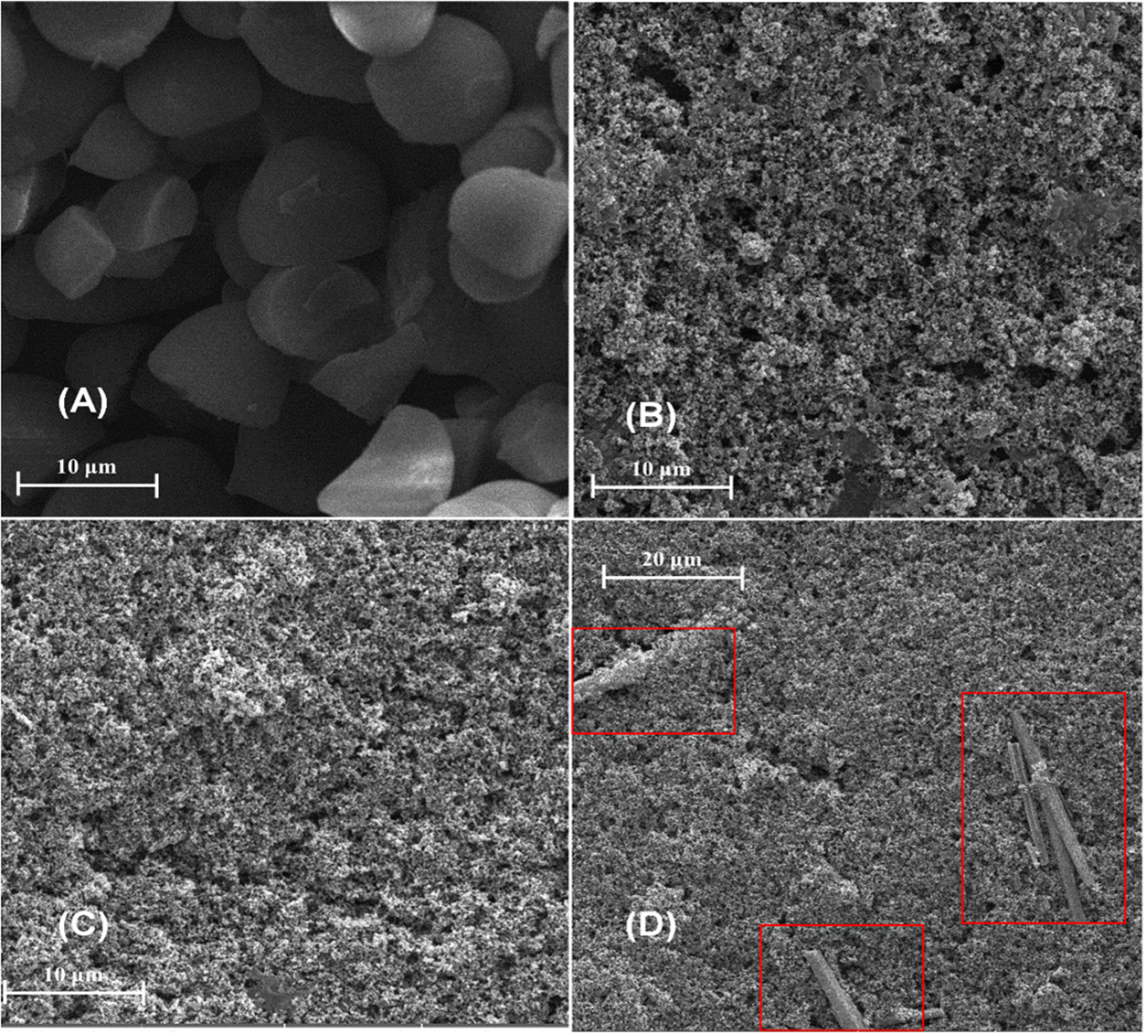
SEM images of the following materials: (A) Starch, (B) Starch/GCE, (C) CBSP/Starch/GCE, and (D) CoPc/CBSP/Starch/GCE.

**Figure 3:**
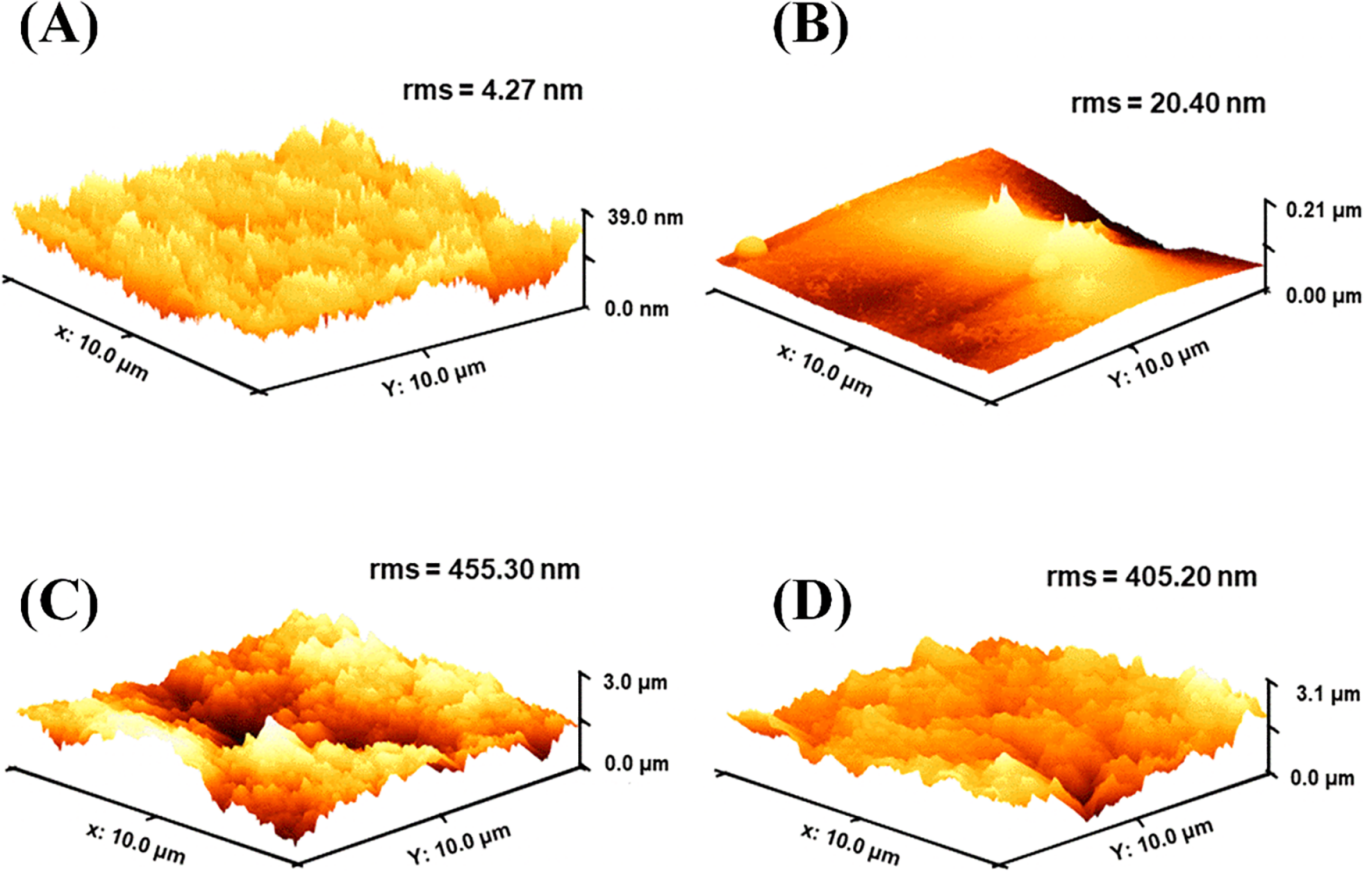
Dispersive AFM images of (A) GCE, (B) Starch/GCE, (C) CBSP/Starch/GCE, and (D) CoPC/CBSP/Starch/GCE.

Figure S2A shows the FTIR spectrum. In blue, we present the spectrum of the starch film, which displays bands at 1300 cm^–1^ associated with C–O vibrations from glucose rings, at 3300 cm^–1^ corresponding to O–H stretching, and at 3000 cm^–1^ related to CH_2_ stretching of the plasticizer (glycerol).^55,56^ For the carbon black spectrum (black line), the band at 1700 cm^–1^ is attributed to the C=O stretching vibration, which is consistent with carbonyl functionalities on the surface of the material. Additionally, the band near 1410 cm^–1^ corresponds to C–H bending vibrations. The band around 1015 cm^–1^ indicates C–O stretching vibrations. Together, the features at ∼1410 cm^–1^ and ∼1300 cm^–1^ remain indicative of sp^2^-hybridized carbon structures typical of graphitic domains.^57,58^ In the presence of phthalocyanine (red line), the bands at 1410 cm^–1^ and 1300 cm^–1^ show increased intensity. In this region, the signals correspond to vibrations of the quinonoid ring and to C–N stretching, respectively. Furthermore, the band at 927 cm^–1^ is assigned to N–H vibrations associated with the phthalocyanine structure.^59,60^

Figure S2B shows the Raman spectra for each modification stage, where the black line illustrates the spectrum for bare GCE. The most distinct signal, around 1577 cm^–1^, known as the G band, corresponds to the in the plane optical vibration mode for two adjacent sp^2^-hybridized carbon atoms in an ideal hexagonal graphite ring. A broader G band indicates a lower degree of structural order. The spectra exhibit a prominent D band centered at 1340 cm^–1^. The presence of the D band is attributed to defects in the curved crystalline structure of carbon. The increased intensity of the D band is associated with a higher structural disorder, resulting from the presence of oxygen-containing functional groups on the surface. Additional combined bands are observed at 2674 cm^–1^ (2D) and 2925 cm^–1^ (D + G). The 2D band arises from a two-photon process involving phonons with opposite wave vectors.^61^ For the starch film on glassy carbon (blue line), a signal similar to that of bare glassy carbon is observed, but with increased noise and a slight shift of the G and D bands. This is due to the strong contribution of Raman signals from the surface layer. If the material in the underlying layer of the sample is less Raman-active, its weak signals will likely be overshadowed by surface noise.^62^ The Raman spectrum profile of CB (red line) is consistent with literature reports, with peaks only for the D and G bands. Compared to the GCE and Starch/GCE, lower overall intensity is observed, although the D and G peaks exhibit comparable relative intensity. The intensity ratio (*I*_D_/*I*_G_) of these two peaks can be used to evaluate the disorder level of carbon materials. Based on this, it is possible to state that the CBSP/Starch/GCE has a lower disorder degree.^63^ For CoPc/CBSP/Starch/GCE (green line), the D and G bands show lower intensity compared to the others, indicating reduced disorder and a lower degree of graphitization in the composite.^64^

To verify the topography of the materials used for the modification of GCE, AFM was used. In Figure 3A, the surface of GCE is shown, where a certain degree of roughness is observed, with the root-mean-square roughness (RMS) equal to 4.27 nm. After adding the starch film (Figure 3B,a) region with protruding peaks, likely resulting from an uneven dispersion via drop-casting, is observed, with an RMS of 20.40. Figure 3C,3D show the topography of CBSP/Starch/GCE and CoPc/CBSP/Starch/GCE, respectively. It is observed that CBSP/Starch/GCE exhibits a higher RMS, likely due to a less uniform dispersion compared to CoPc/CBSP/Starch/GCE. However, in general, both surfaces exhibit similar roughness values, with a variation of approximately 10%. The incorporation of CoPc tends to promote a more homogeneous dispersion of conductive carbon aggregates within the starch film due to π–π interactions between the macrocyclic aromatic structure of CoPc and the sp^2^-carbon domains of CB.^65,66^ Comparing Figure 3A,3D, an approximately 100-fold increase in RMS is observed, which can indicate an increase in effective surface area favored by the modifier.

### 3.2 Modifier Composition

The electrochemical response to the 1.0 mmol L^–1^ [Fe­(CN)_6_]^3–^ probe in 0.5 mol L^–1^ KCl was first assessed on the GCE modified with a starch film (Starch/GCE), in the presence and absence of 5% acetic acid in the film composition. This evaluation aimed to determine the optimal composition of the starch film extracted from the mesocarp of the babassu coconut for application in an electrochemical sensor. Figure S3A shows that Starch/GCE with acetic acid has sharper peaks and a smaller ΔE_P_ compared to Starch/GCE without acetic acid. This suggests that the presence of ions in the gelatinized starch matrix increases electrical conductivity.^67^ A study was undertaken to establish the optimal film concentration of the starch film, ranging from 25 to 100 mg L^–1^. No significant alterations in the redox profile of the probe were observed, as shown in Figure S3B. Therefore, a concentration of 50 mg L^–1^ of starch was selected for use.

Figure S3C shows the cyclic voltammograms of the redox probe 1.0 mmol L^–1^ [Fe­(CN)_6_]^3–/4–^ in 0.5 mol L^–1^ KCl solution on GCE (black line), Starch/GCE (red line), CBSP/Starch/GCE (indigo line), and CoPc/CBSP/Starch/GCE (blue line). Table S1 clearly shows that GCE and Starch/GCE exhibit lower *I*_PC_ values and a bigger separation between the probe’s redox couples (Δ*E*_P_). However, for CBSP/Starch/GCE and CoPc/CBSP/Starch/GCE, the redox pair separation (Δ*E*_P_) was smaller, closer to the predicted value for reversible systems involving a single-electron transfer.^68^ Furthermore, the cathodic peak current (*I*_PC_) values increased. This is consistent with the literature, which reports that the addition of CBSP increases porosity, thereby increasing the conductivity of the film.^10^ Likewise, the incorporation of CoPc not only significantly increases peak current but also reduces the peak potential, indicating an improvement in the kinetic parameters.^69^

The EIS experiments were performed to evaluate the charge transfer resistance (*R*_ct_) (Figure S4). It was observed that the addition of the starch film increases the resistance compared to the bare GCE, as evidenced by the larger semicircle in the Nyquist plot of Starch/GCE. When comparing the *R*_ct_ values obtained through an adapted Randles circuit, values of 6.04 and 15.6 kΩ were found for bare GCE and Starch/GCE, respectively. A significant decrease in *R*_ct_ is observed for CBSP/Starch/GCE and CoPc/CBSP/Starch/GCE, with values of 39 and 18 Ω, respectively, compared to the other two diagrams. The sp^2^-hybridized carbon structure and porous nanoaggregate morphology of CBSP significantly enhance the electronic percolation pathways within the composite, leading to higher peak currents and a reduced Δ*E*_p_ for the [Fe­(CN)_6_]^3–^/^4–^ redox probe when compared to bare GCE and Starch/GCE.^70^ This improvement is quantitatively supported by EIS results, which show a drastic decrease in charge-transfer resistance from the kΩ range (GCE and Starch/GCE) to 39 Ω for CBSP/Starch/GCE. The further incorporation of CoPc amplifies these effects, reducing *R*_ct_ to 18 Ω and confirming the synergistic contribution of the electroactive CoPc layer and the conductive CBSP network to the enhanced electron-transfer kinetics.

### 3.3 Electrochemical Behavior of HCQ

Cyclic voltammetry (Figure 4A) was used to investigate the electrochemical behavior of 20 μmol L^–1^ HCQ in 0.1 mol L^–1^ phosphate buffer using GCE before and after different surface modifications. In the voltammograms, two characteristic oxidation peaks of HCQ can be observed at approximately +0.80 V (vs Ag|AgCl|KCl­(sat.)) (Peak 1) and +1.05 V (Peak 2), in agreement with previous reports. These two oxidation processes are associated with the stepwise oxidation of the HCQ molecule, in which the main irreversible oxidation at higher potential is related to the tertiary amine group through a proton-coupled electron transfer process. As illustrated in the proposed reaction pathway (Figure 4B), this process involves a two-electron and two-proton (2e^–^/2H^+^) oxidation mechanism.^71^

**Figure 4:**
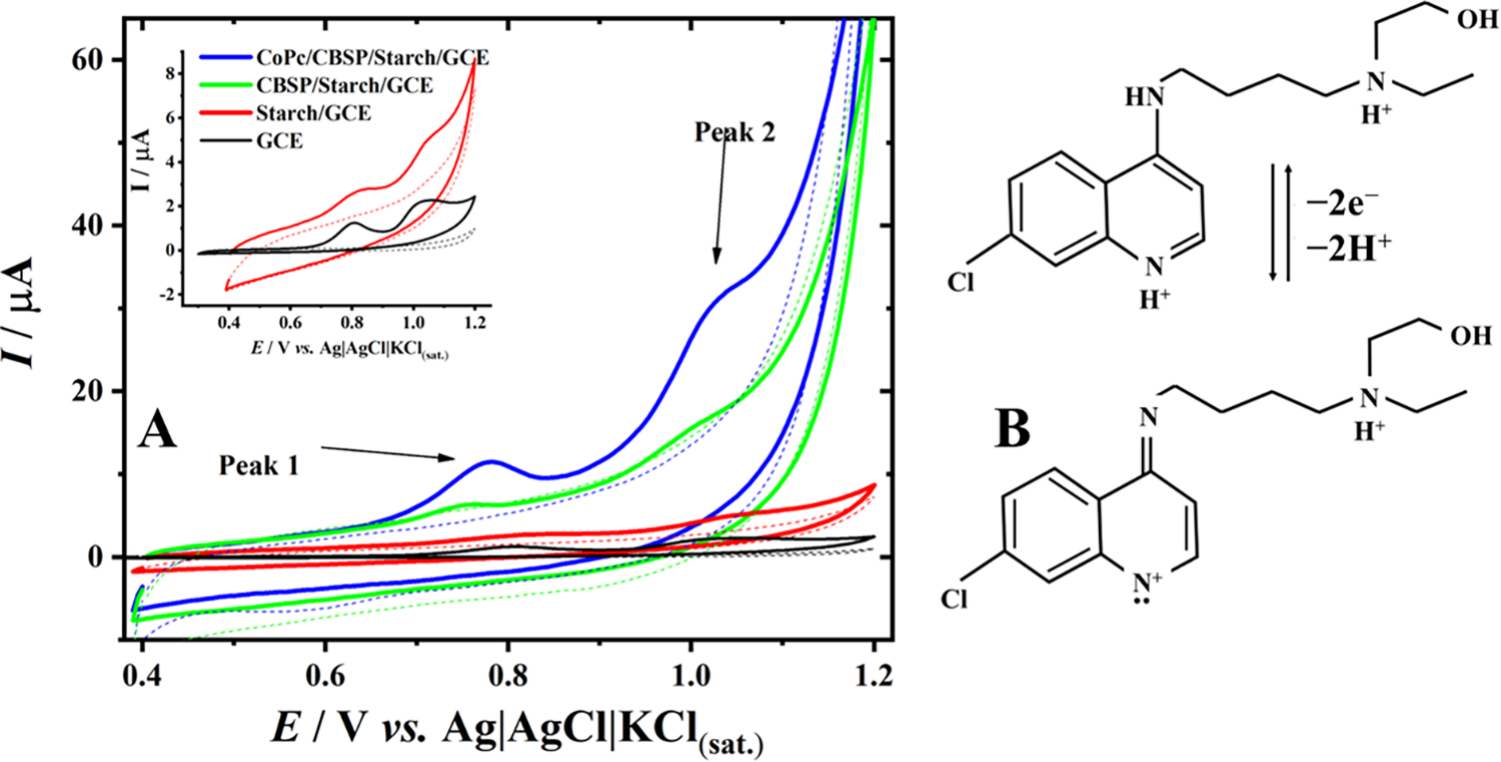
(A**)** CV in HCQ (10 μmol L^–1^) in 0.1 mol L^–1^ Phosphate Buffer (pH 7.0), on GCE (black), Starch/GCE (red line), CBSP/Starch/GCE (green line), and CoPc/CBSP/Starch/GCE (blue line) at *E*_step_ = 5 mV; 50 mV s^–1^; *T*_PC_ = 20 s. (B) Schematic representation of electrochemical oxidation of hydroxychloroquine.

Both the capacitive current, arising from the electric double-layer contribution typical of carbon black-based electrodes, and the faradaic analytical signal are significantly enhanced at the CoPc/CBSP/Starch/GCE. The oxidation currents recorded at the bare GCE were 0.69 μA (IP1) and 0.73 μA (IP2), whereas at the CoPc/CBSP/Starch/GCE they increased to 3.90 μA (IP1) and 3.22 μA (IP2), corresponding to approximately 6-fold and 5-fold signal enhancements, respectively. This pronounced increase in current, together with the lower onset potential observed for the modified electrode, confirms the electrocatalytic role of cobalt phthalocyanine. In this system, CoPc acts as a redox mediator through the Co­(II)/Co­(III) couple, facilitating electron transfer from HCQ to the electrode surface via an EC’ catalytic mechanism, while carbon black enhances charge transport and electroactive area and starch promotes analyte adsorption.^72,73^ The π–π stacking interaction between CoPc and the sp^2^-hybridized domains of carbon black promotes charge delocalization at their interface, facilitating faster electron injection from the CoPc active sites into the conductive carbon network.^70,74^ This enhanced electronic coupling reduces interfacial resistance, leading to higher catalytic currents and shifting the oxidation potential of hydroxychloroquine to less positive values. This catalytic mechanism lowers the overpotential and accelerates the interfacial charge-transfer kinetics, which is experimentally reflected by the increased anodic peak current and the shift of the oxidation potential to less positive values.

The electrochemical results demonstrate a synergistic contribution from starch, carbon black, and CoPc toward the oxidation of hydroxychloroquine. The starch film provides an ion-conducting and hydrophilic matrix that stabilizes the dispersion of CBSP and CoPc, while carbon black introduces a highly conductive sp^2^-carbon network that enhances charge-transport pathways, reflected by the reduced *R*_ct_ and Δ*E*_p_. Together, these components accelerate electron-transfer kinetics and strengthen analyte–electrode interactions, accounting for the superior catalytic current and lower detection limits achieved with the CoPc/CBSP/Starch/GCE electrode. As a result, the improved electron-transfer efficiency and enhanced analyte–catalyst interactions lead to higher sensitivity and lower detection limits.

### 3.4 pH, Supporting Electrolyte and Scan Rate Study

A study was conducted by CV technique to evaluate the electrochemical response of 10 μmol L^–1^ HCQ in 0.1 mol L^–1^ B-R buffer over a pH range of 6.0 to 10.0. The goal was to examine the effect of pH on the electrochemical response of HCQ at the modified film and to identify the optimal medium for its electrochemical detection. Figure S5 demonstrates that a neutral medium (pH 7.0) yields the highest current response (IP1) for HCQ, with reduced peak intensities observed at other pH values. A decrease followed by an increase in current within the pH range of 8.0–9.0 is also observed, which can be attributed to the p*K*_a_ of HCQ (p*K*_a_ = 8.3), indicating that the molecule remains protonated at pH 7.4.^75^ The oxidation potential values (*E*_P1_) shifted linearly to more negative values as the pH increased. As shown in Figure S5B, the slope and intersection coefficient of the pH vs *E*_P1_ plot support a linear relationship, consistent with the extended form of the Nernst equation.^76^ The resulting equation is $E_{P1}=-0.073pH+1.31\;\left(R^{2}=0.999\right)$ . Therefore, the peak potential shifts at a rate of 0.073 V pH^–1^, indicating that the electrochemical oxidation of HCQ involves an equal number of protons and electrons (H^+^/e^–^), as predicted by the Nernst equation.^76^

To find the optimal supporting electrolyte for HCQ determination, three solutions were evaluated: 0.1 mol L^–1^KCl, 0.1 mol L^–1^ PB (pH 7.0), and B-R buffer (pH 7.0). CV tests were performed in triplicate in the three different mediums using 10 μmol L^–1^ HCQ. Figure S6 shows the corresponding voltammograms and compares the peak current values (*I*_P1_) for each supporting electrolyte [65]. Among the tested electrolytes, both PB and the B-R buffer produced the best results for HCQ analysis, providing well-defined peaks and appreciable current responses (*I*_P1_). However, PB outperformed the others, exhibiting the highest current signal.

The effect of the scan rate was studied to evaluate the contribution of the diffusion and adsorption processes and to determine which mechanism predominates in the electrochemical reaction. This study was conducted using CV in 0.1 mol L^–1^ PB (pH 7.0), with scan rates ranging from 10 to 150 mV s^–1^. Figure S7A shows the voltammograms obtained during the scan rate experiment, where an increase in peak current is observed with increasing scan rate. This behavior is attributed to the reduction in the diffusion layer thickness at higher scan rates.^77^ Figure S7B, in turn, shows the relationship between scan rate (ν, mV s^–1^) and the peak current (*I*_P1_, μA), where a good linearity is observed, based on the correlation coefficient (*R* ^2^ = 0.998). Figure S7C shows *R* ^2^ of 0.968 between ν^1^/^2^ vs *I*_P1_, indicating that the process is adsorption-controlled. To further clarify the transport mechanism of the electroactive species, a log–log plot of peak current (Log IP) versus scan rate (Log ν) was also constructed (Figure S7D), based on the power-law dependence of current on scan rate.^77^ Since the obtained coefficient was 0.94, it can be concluded that the electronic charge transfer process is adsorptive in nature. From the scan rate study (Figure S7E), it is also possible to predict the number of electrons involved in the reaction for each redox signal using Laviron’s equation.^77^ From the first peak, an n value of 1.5 was calculated, which can be interpreted as involving two electrons in the electrochemical reaction, similar to cases reported in the literature.^78–80^

### 3.5 Fouling Effects on Electrode Performance

The electrocatalytic interface of solid electrodes is generally prone to “fouling”, depending on the nature of the analyte, and consecutive scans often fail to yield reproducible results.^81^ To evaluate the antifouling effect of the film on the electrode surface, nine cyclic voltammetric scans were intermittently performed in a 0.1 mol L^–1^ PB solution containing 20 μmol L^–1^ HCQ on both the bare GCE and the CoPc/CBSP/Starch/GCE. Figure 5A shows the scans performed with the bare GCE, where a gradual and significant decrease of 70.5% in peak current (*I*_P1_) is observed when comparing the first to the last cycle. Additionally, there is a positive shift of 80 mV in the oxidation potential (*E*_P1_). Conversely, the CoPc/CBSP/Starch/GCE showed an 8.5% fluctuation in the peak current (*I*_P1_) (Figure 5B), which was mostly attributed to the inherent standard deviation of the method itself, as it has not yet been fully optimized at this stage of the process. It was also noted that there was no shift in the oxidation potential for the CoPc/CBSP/Starch/GCE, indicating that the electrode–solution interface remained unchanged.

**Figure 5:**
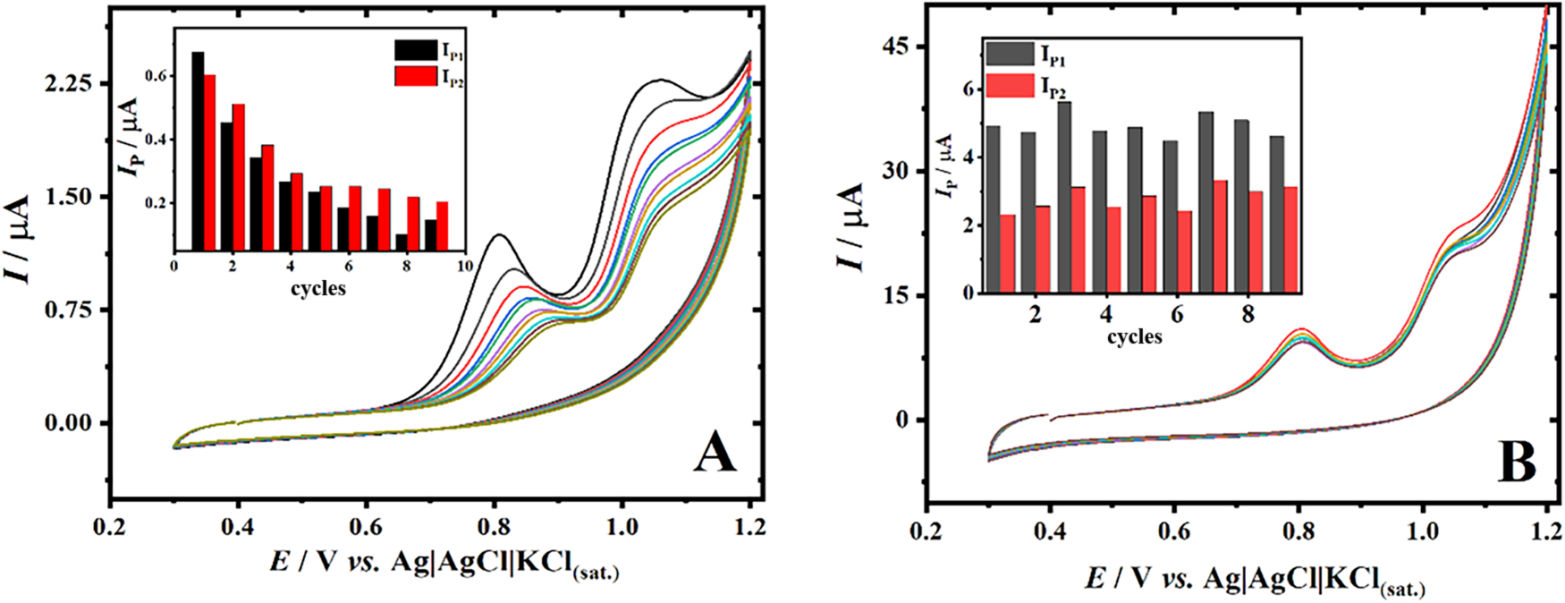
CV in the presence of HCQ (20 μmol L^–1^) in 0.1 mol L^–1^ PB (pH 7.0), on (A) bare GCE and (B) CoPc/CBSP/Starch/GCE. *n* = 9, *E*_step_ = 5 mV; scan rate = 50 mV s^–1^; *T*_PC_ = 20 s.

### 3.6 Calibration Curve, Repeatability, and Determination of HCQ in Samples

Given the optimized method for determining HCQ, linear sweep voltammetry (LSV) was performed in the potential range of 0.4 to 1.2 V, with a scan rate of 100 mV s^–1^, and using 0.1 mol L^–1^ PB (pH 7.0) as the supporting electrolyte. Although pulsed techniques like differential pulse voltammetry (DPV) offer higher sensitivity, linear sweep voltammetry (LSV) was chosen for its faster acquisition time and greater stability in adsorption-controlled processes, such as those involved in HCQ analysis.^82^ Therefore, LSV was selected for the determination of HCQ. Additionally, the analyzed concentration range did not require the ultralow detection limits of pulsed techniques, making LSV a suitable choice for hydroxychloroquine determination under the optimized conditions. Successive additions of HCQ were made to generate a concentration range from 0.1 to 16.0 μmol L^–1^ for the construction of a calibration curve and to assess the linearity of the method. The current response obtained for Peak 1 was used to construct the calibration curve shown in Figure 6. Linearity was observed across the entire concentration range, with an *R* ^2^ of 0.999, yielding the following equation: *I*_P1_(μA) = 1.25 ± 6.55 *C*_HCQ_ (μmol L^–1^) + 0.70 ± 8.92. Using the IUPAC definition, the limits of detection (LOD) and quantification (LOQ) were calculated using the equations: LOD = 3*S*(*y*/*x*)/*b* and LOQ = 10*S*(*y*/*x*)/*b*, where *S*(*x*/*y*) is the residual standard deviation and b is the slope of the calibration curve. Thus, LOD = 0.015 μmol L^–1^ and LOQ = 0.052 μmol L^–1^ were obtained. As reported in the literature, HCQ concentrations detected in water samples from countries such as Nigeria, the United States of America, Greece, and Spain reached levels of 0.15 μmol L^–1^ HCQ.^83^ The present method demonstrates good sensitivity and effectiveness for determining HCQ in real river water samples, as its detection limit is lower than the concentrations reported in multiple case studies.

**Figure 6:**
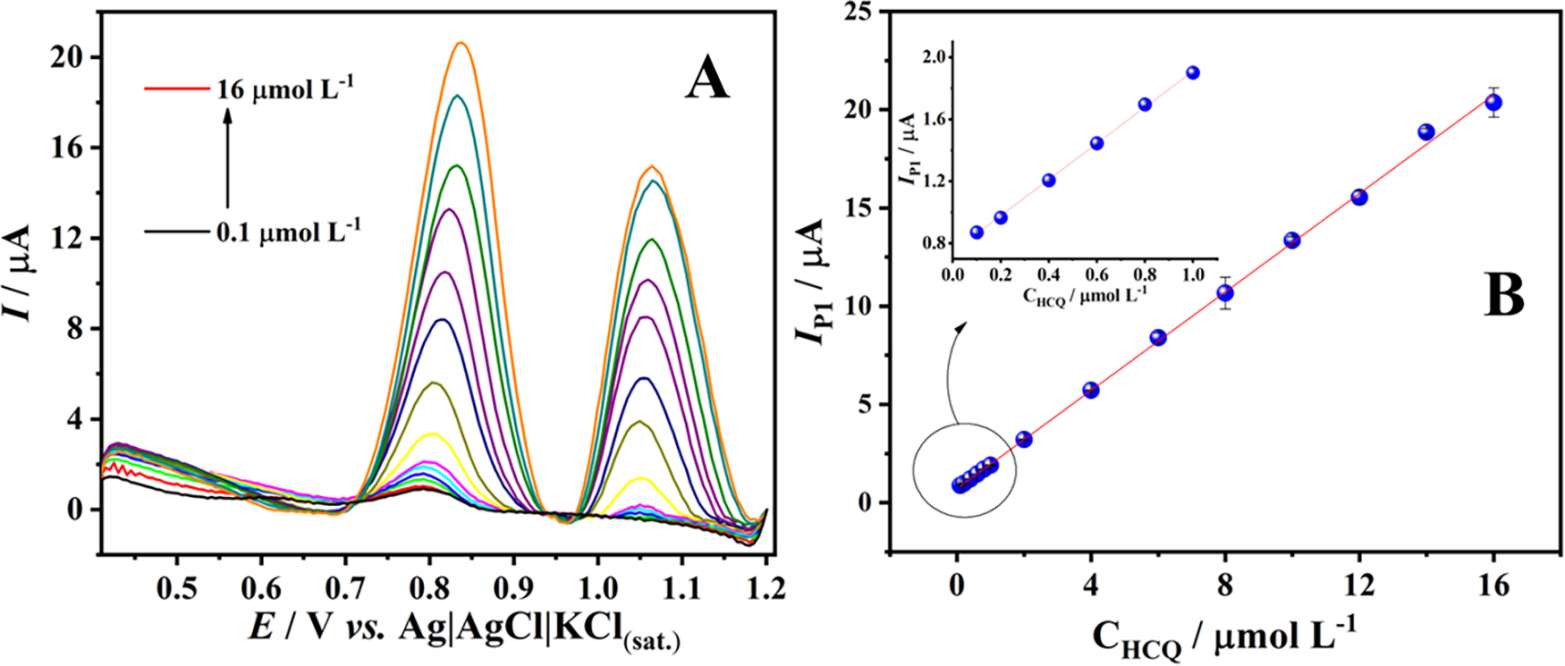
(A) LSV (baseline corrected) of CoPc/CBSP/Starch/GCE in PB 0.1 mol L^–1^ (pH 7.0) in the presence of HCQ (0.1–16.0 μmol L^–1^). San rate = 100 mV s^–1^; *E*_step_ = 5 mV; *T*_PC_ = 2 min. (B) Peak current (Ip) dependence on HCQ concentration (μmol L^–1^).

To assess the intraelectrode precision of the method, relative standard deviations (RSDs) were calculated for the first peak current (*I*_P1_), yielding values of 3%, 2%, and 1% for HCQ concentrations of 0.5, 5.0, and 10.0 μmol L^–1^, respectively (Figure S8). These results demonstrate the antifouling effect of the film, as low RSD values were maintained even at higher concentrations after several consecutive scans performed intermittently. It is important to highlight that the same electrode was used for all measurements across the three concentration levels. For interelectrode precision, the RSD was calculated for the voltammetric response of three independently prepared electrodes (*n* = 3), resulting in a value of 6% for an HCQ concentration of 5.0 μmol L^–1^. This is considered acceptable given that drop-casting was used as the electrode preparation technique, which can inherently introduce variability in application.

Subsequently, an interference study was carried out for the CoPc/CBSP/Starch/GCE in the presence of possible interfering substances (Figure S10). The salts CaCl_2_, KCl, Na_2_HPO_4_, Na_2_SO_4_, and NaNO_3_ were selected as interfering species because they represent the main cations and anions commonly found in natural and potable waters, such as Ca^2+^, K^+^, Na^+^, Cl^–^, HPO_4_^2–^, SO_4_^2–^, and NO_3_^–^.^84,85^ These ions are directly associated with water hardness, alkalinity, salinity, and nutrient content in aquatic systems.^86^ Considering that the matrices employed in the addition and recovery assays were river water and tap water samples, the selection of these species allowed a realistic simulation of the ionic composition of these matrices and, consequently, a reliable evaluation of the method selectivity toward relevant ionic interferences.^87^ Under a HCQ/interferent ratio of 1:1000, in all evaluated cases, the signal variation did not exceed 6%, demonstrating the high selectivity and robustness of the proposed method even under conditions of significant excess of potentially interfering ions.

The operational stability of the fabricated electrode was evaluated by recording 50 successive linear voltammetric measurements using the same electrode (Figure S11). The relative standard deviation (RSD) of the peak current obtained from these measurements was 7.2%, indicating good repeatability and satisfactory signal stability over consecutive scans. This result demonstrates that the electrode maintains its electrochemical performance under continuous operation, with no significant loss of sensitivity or surface fouling throughout the measurements. Therefore, the proposed sensor exhibits adequate stability for reliable analytical applications.

To demonstrate the applicability of the proposed method, tests were conducted on three different real sample matrices: tap water, pharmaceutical samples, and river water. The study was carried out with the first oxidation peak of the molecule (EP_1_), using the standard addition method. Two fortification levels were applied to each matrix, within the concentration range of 0.1–1.0 μmol L^–1^, as shown in Figure S9. Table 1 presents the addition and recovery results for each sample, showing recovery percentages ranging from 100% to 105%. These values indicate satisfactory recovery, especially considering the complexity of environmental samples, which may contain various interfering substances such as chemical pollutants, colloidal particles, bacteria, and others.^88^ For pharmaceutical samples, the presence of tablet excipients, which can act as potential interferents in electroanalytical analysis, was also taken into account. Additionally, the low RSD observed indicate good precision of the method.

**Table 1:** Recovery Values for Tap Water, Pharmaceutical Tablet, and River Water Samples

sample	spiked/μmol L^–1^	found/μmol L^–1^	recovery/%
Tap water A	0.0	<LOD	----
	0.20^a^	0.206 (±0.01)	103 (±1)
Tap water B	0.0	<LOD	----
	1.00^a^	1.018 (±0.07)	102 (±7)
River water A	0.0	<LOD	----
	0.10^a^	0.100 (±0.03)	100 (±3)
River water B	0.0	<LOD	----
	0.20^a^	0.209 (±0.02)	104 (±2)
Tablet A	0.50^b^	0.526 (±0.06)	105 (±6)
Tablet B	1.00^b^	1.036 (±0.08)	104 (±8)

a Concentration after spiking with HCQ standard.

b Concentration after drug dilution based on the label information on the pharmaceutical packaging.

Notably, our LOD remains below the average reported in recent literature, demonstrating that competitive performance can be attained without intricate synthesis or expensive components. Although some reported sensors achieve lower detection limits (LOD) for hydroxychloroquine (HCQ) than our proposed method (Table S2), our system offers a compelling balance between sensitivity, cost-effectiveness, and simplicity. While advanced nanomaterials or complex electrode modifications (graphene hybrids, metal–organic frameworks) may yield superior LODs, our sensor relies on low-cost materials and a straightforward modification process. Furthermore, the use of LSV a simpler technique compared to pulse-based methods highlights the method’s practicality for routine analysis. While ultrasensitive detection is critical for trace-level applications, our sensor targets clinically or environmentally relevant HCQ concentrations, where excessive sensitivity may be unnecessary.

## 4 Conclusions

In this study, the efficiency of Carbon Black Super P and Cobalt Phthalocyanine as modifiers in the electrode for the determination of hydroxychloroquine was verified using a low-cost and simple preparation technique. The number of protons in the electrochemical oxidation of HCQ is equal to the number of electrons, and its mass transport process to the modified electrode has an adsorptive nature. The starch film provided protection against electrode passivation and ensured mechanical stability for the modifier on the electrode surface. The CoPc/CBSP/Starch/GCE showed an approximately 10-fold increase in electroactive area compared to the bare GCE, which also resulted in a higher current signal. The modifier promoted both physical and chemical enhancements, leading to increased current, enlarged electroactive area, as well as adsorptive properties that enable a simple and efficient preconcentration step. Under optimal analysis conditions, the LOD and LOQ were 0.015 μmol L^–1^ and 0.052 μmol L^–1^, respectively. The method also showed excellent linearity in the range of 0.1 to 16.0 μmol L^–1^ (*r* ^2^ = 0.998). The accuracy, evaluated through the addition and recovery method, indicates the method’s suitability, especially for the determination of HCQ in water from treatment stations. Given the results, the reliability and effectiveness of the method for both detection and quantification of HCQ are evident. Considering the context in which the study was developed, it is also important to highlight its social and environmental relevance aspect.

## Supplementary Material



## Notes

EDS analysis, Raman and FTIR spectra, cyclic voltammograms related to the optimization studies (modifier concentration, pH, supporting electrolyte, and scan rate), repeatability results, interference and electrode stability studies, electrochemical impedance spectroscopy data, and application of the proposed sensor to real samples; Tables comparing the modifiers and benchmarking the analytical performance of the proposed sensor against previously published literature (PDF)

## References

[1] Devi N., Kumar R., Singh S., Singh R. K. (2024). Recent Development
of Graphene-Based Composite for Multifunctional Applications: Energy,
Environmental and Biomedical Sciences. Crit.
Rev. Solid State Mater. Sci..

[2] Baig N., Saleh T. A. (2018). Electrodes Modified
with 3D Graphene Composites: A
Review on Methods for Preparation, Properties and Sensing Applications. Microchim. Acta.

[3] Hindermann-Bischoff M., Ehrburger-Dolle F. (2001). Electrical
Conductivity of Carbon Black–Polyethylene
Composites. Carbon.

[4] Tomczyk D., Seliger P. (2024). Modification of Glassy
Carbon Electrodes with Complexes
of Manganese­(II) with Some Phenanthroline Derivatives Immobilized
in Nafion Layer. Int. J. Mol. Sci..

[5] Dilgin D. G., Vural K., Karakaya S., Dilgin Y. (2024). Simple, Sensitive,
and Cost-Effective Voltammetric Determination of Salbutamol at a Pencil
Graphite Electrode Modified with Nafion and Functionalized Multi-Walled
Carbon Nanotubes. Monatsh. Chem.—Chem.
Monthly.

[6] Deroco P. B., Melo I. G., Silva L. S. R., Eguiluz K. I. B., Salazar-Banda G. R., Fatibello-Filho O. (2018). Carbon Black
Supported Au–Pd Core-Shell Nanoparticles
within a Dihexadecylphosphate Film for the Development of Hydrazine
Electrochemical Sensor. Sens. Actuators, B.

[7] Khaled N. I., Santhiya D. (2024). Multifunctional Poly­(Allylamine
Hydrochloride)/Bioactive
Glass Layer by Layer Surface Coating on Magnesium Alloy for Biomedical
Applications. Prog. Org. Coat..

[8] Ping J., Wu J., Luo X., Ying Y. (2011). The Use of the Platinum Electrode
Coated with Ultrathin Poly­(Allylamine Hydrochloride)/Nafion Films
for Selective Detection of Hydrogen Peroxide. Ionics.

[9] Xie F. (2024). Natural Polymer
Starch-Based Materials for Flexible Electronic Sensor Development:
A Review of Recent Progress. Carbohydr. Polym..

[10] Vicentini F. C., Silva L. R. G., Stefano J. S., Lima A. R. F., Prakash J., Bonacin J. A., Janegitz B. C. (2023). Starch-Based
Electrochemical Sensors
and Biosensors: A Review. Biomed. Mater. Devices.

[11] Zou J., Chen X.-Q., Zhao G.-Q., Jiang X.-Y., Jiao F.-P., Yu J.-G. (2019). A Novel
Electrochemical Chiral Interface Based on the Synergistic
Effect of Polysaccharides for the Recognition of Tyrosine Enantiomers. Talanta.

[12] Camargo J. R., Baccarin M., Raymundo-Pereira P. A., Campos A. M., Oliveira G. G., Fatibello-Filho O., Oliveira O. N., Janegitz B. C. (2018). Electrochemical
Biosensor Made with Tyrosinase Immobilized in a Matrix of Nanodiamonds
and Potato Starch for Detecting Phenolic Compounds. Anal. Chim. Acta.

[13] Orzari L. O., Santos F. A., Janegitz B. C. (2018). Manioc Starch Thin
Film as Support
of Reduced Graphene Oxide: A Novel Architecture for Electrochemical
Sensors. J. Electroanal. Chem..

[14] Gao R., Guo W., Zhang Y., Zhang Q., Li Y., Wang J. (2024). Enhancement
of Gelatinization on Electrochemical Performance of Corn Starch-Based
Porous Carbon as Electrode Material in Supercapacitors. Diamond Relat. Mater..

[15] Borges L. A., Ramos K. K., Felisberto M. H. F., Franciosi E. R. N., Efraim P. (2023). Babassu Mesocarp: A
Sustainable Source for Obtaining
Starch and New Products. Starch - Stärke.

[16] Souza M. H. S. L., Monteiro C. A., Figueredo P. M. S., Nascimento F. R. F., Guerra R. N. M. (2011). Ethnopharmacological Use of Babassu (Orbignya Phalerata
Mart) in Communities of Babassu Nut Breakers in Maranhão, Brazil. J. Ethnopharmacol..

[17] Lima R. C., de Carvalho A. P. A., de Almeida A. E. C. C., Conte-Junior C. A. (2024). Bioactive
Compounds and Benefits of By-Products of Amazon Babassu Oil Production:
Potential for Dietary Supplement, Biomedical and Food Applications. Food Funct.

[18] Cinelli B. A., López J. A., Castilho L. R., Freire D. M. G., Castro A. M. (2014). Granular
Starch Hydrolysis of Babassu Agroindustrial Residue: A Bioprocess
within the Context of Biorefinery. Fuel.

[19] Lima H. R. S., Airton de Oliveira Farias E., Teixeira P. R. S., Eiras C., Nunes L. C. C. (2019). Blend Films Based
on Biopolymers Extracted from Babassu
Mesocarp (Orbignya Phalerata) for the Electrochemical Detection of
Methotrexate Antineoplastic Drug. J. Solid State
Electrochem..

[20] Brainina Kh., Stozhko N., Bukharinova M., Vikulova E. (2018). Nanomaterials: Electrochemical
Properties and Application in Sensors. Phys.
Sci. Rev..

[21] Mansur W., Shoukat R., Kaleli M., Akyürekli S., Ahmad M., Ahmed A. Y., Ali A. (2025). A Surface Modified
Carbon Nanotube Fiber as a Microelectrode for the PH-Dependent Electrochemical
Detection of Vitamin C. ACS Omega.

[22] Zia R., Ahmad M., Kaleem M., Akyürekli S., Hassan S. U., Mohammed O. A., Alzahrani F. M., Iqbal M., Ali A. (2025). Polyoxometalate Decorated
Graphene
Oxides Sheets for Simultaneous Non-Enzymatic Electrochemical Detection
of Biomolecules. Diamond Relat. Mater..

[23] Nazir A., Muqaddas S., Ali A., Jamshaid T., Riaz S., Iqbal M., Kaleli M., Akyürekli S., Naeem H., Mahmoud H. M. A., Ahmed A. M. E. (2024). A Flexible
Carbon
Nanotubes-Based Microelectrode for Non-Enzymatic Electrochemical Uric
Acid and Ascorbic Acid Sensing. Mater. Sci.
Eng.: B.

[24] Muqaddas S., Javed M., Nadeem S., Asghar M. A., Haider A., Ahmad M., Ashraf A. R., Nazir A., Iqbal M., Alwadai N., Ahmad A., Ali A. (2023). Carbon Nanotube Fiber-Based
Flexible Microelectrode for Electrochemical Glucose Sensors. ACS Omega.

[25] Yang J.-H., Gao Y., Zhang W., Tang P., Tan J., Lu A.-H., Ma D. (2013). Cobalt Phthalocyanine–Graphene
Oxide Nanocomposite: Complicated
Mutual Electronic Interaction. J. Phys. Chem.
C.

[26] Silva T. A., Moraes F. C., Janegitz B. C., Fatibello-Filho O. (2017). Electrochemical
Biosensors Based on Nanostructured Carbon Black: A Review. J. Nanomater..

[27] Wang M., Torbensen K., Salvatore D., Ren S., Joulié D., Dumoulin F., Mendoza D., Lassalle-Kaiser B., Işci U., Berlinguette C. P., Robert M. (2019). CO2 Electrochemical
Catalytic Reduction with a Highly Active Cobalt Phthalocyanine. Nat. Commun..

[28] Della
Pelle F., Angelini C., Sergi M., Del Carlo M., Pepe A., Compagnone D. (2018). Nano Carbon Black-Based Screen Printed
Sensor for Carbofuran, Isoprocarb, Carbaryl and Fenobucarb Detection:
Application to Grain Samples. Talanta.

[29] An
Wong C. H., Ambrosi A., Pumera M. (2012). Thermally Reduced Graphenes
Exhibiting a Close Relationship to Amorphous Carbon. Nanoscale.

[30] Vicentini F. C., Raymundo-Pereira P. A., Janegitz B. C., Machado S. A. S., Fatibello-Filho O. (2016). Nanostructured
Carbon Black for Simultaneous Sensing in Biological Fluids. Sens. Actuators, B.

[31] Lo T. W. B., Aldous L., Compton R. G. (2012). The Use of Nano-Carbon
as an Alternative
to Multi-Walled Carbon Nanotubes in Modified Electrodes for Adsorptive
Stripping Voltammetry. Sens. Actuators, B.

[32] Medalia A. I., Heckman F. A. (1969). Morphology of Aggregates—II. Size and Shape
Factors of Carbon Black Aggregates from Electron Microscopy. Carbon.

[33] Chowdhury Z. Z., Sagadevan S., Bin Johan R., Shah S. T., Adebesi A., Islam Md S., Rafique R. F. (2018). A Review
on Electrochemically Modified
Carbon Nanotubes (CNTs) Membrane for Desalination and Purification
of Water. Mater. Res. Express.

[34] Porto L. S., da Silva D. N., Silva M. C., Pereira A. C. (2019). Electrochemical
Sensor Based on Multi-walled Carbon Nanotubes and Cobalt Phthalocyanine
Composite for Pyridoxine Determination. Electroanalysis.

[35] Wring S. A., Hart J. P., Bracey L., Birch B. J. (1990). Development of Screen-Printed
Carbon Electrodes, Chemically Modified with Cobalt Phthalocyanine,
for Electrochemical Sensor Applications. Anal.
Chim. Acta.

[36] Kunpatee K., Chamsai P., Mehmeti E., Stankovic D. M., Ortner A., Kalcher K., Samphao A. (2019). A Highly Sensitive
Fenobucarb Electrochemical Sensor Based on Graphene Nanoribbons-Ionic
Liquid-Cobalt Phthalocyanine Composites Modified on Screen-Printed
Carbon Electrode Coupled with a Flow Injection Analysis. J. Electroanal. Chem..

[37] Chaiyo S., Mehmeti E., Siangproh W., Hoang T. L., Nguyen H. P., Chailapakul O., Kalcher K. (2018). Non-Enzymatic Electrochemical Detection
of Glucose with a Disposable Paper-Based Sensor Using a Cobalt Phthalocyanine–Ionic
Liquid–Graphene Composite. Biosens. Bioelectron..

[38] Demir E., Silah H., Uslu B. (2022). Phthalocyanine
Modified Electrodes
in Electrochemical Analysis. Crit. Rev. Anal.
Chem..

[39] Mohammed I., Nemakal M., Aralekallu S., Sajjan V. A., Divakara T. R., Palanna M., Keshavananda
Prabu C.
P., Sannegowda L. K. (2020). Phthalocyanine
Sheet Polymer Based Amperometric Sensor for the Selective Detection
of 2,4-Dichlorophenol. J. Electroanal. Chem..

[40] Prabhu
C P K., Aralekallu S., Sannegowda L. K. (2024). Efficacy of Phthalocyanine-Based
Catalysts in Electrochemical Sensors: A Comprehensive Review. Adv. Sens. Res..

[41] Kuntoji G., Kousar N., Gaddimath S., Sannegowda L. K. (2024). Macromolecule–Nanoparticle-Based
Hybrid Materials for Biosensor Applications. Biosensors.

[42] Ben-Zvi I., Kivity S., Langevitz P., Shoenfeld Y. (2012). Hydroxychloroquine:
From Malaria to Autoimmunity. Clin. Rev. Allergy
Immunol..

[43] Liu J., Cao R., Xu M., Wang X., Zhang H., Hu H., Li Y., Hu Z., Zhong W., Wang M. (2020). Hydroxychloroquine,
a Less Toxic Derivative of Chloroquine, Is Effective in Inhibiting
SARS-CoV-2 Infection in Vitro. Cell Discovery.

[44] El
Mhammedi M. A., Saqrane S., Lahrich S., Laghrib F., El Bouabi Y., Farahi A., Bakasse M. (2020). Current Trends in Analytical
Methods for the Determination of Hydroxychloroquine and Its Application
as Treatment for COVID-19. ChemistrySelect.

[45] Urban R. C., Nakada L. Y. K. (2021). COVID-19 Pandemic: Solid Waste and Environmental Impacts
in Brazil. Sci. Total Environ..

[46] da
Luz T. M., Araújo A. P., da C., Estrela F. N., Braz H. L. B., Jorge R. J. B., Charlie-Silva I., Malafaia G. (2021). Can Use of Hydroxychloroquine and Azithromycin as a
Treatment of COVID-19 Affect Aquatic Wildlife? A Study Conducted with
Neotropical Tadpole. Sci. Total Environ..

[47] Kargar F., Bemani A., Sayadi M. H., Ahmadpour N. (2021). Synthesis
of Modified Beta Bismuth Oxide by Titanium Oxide and Highly Efficient
Solar Photocatalytic Properties on Hydroxychloroquine Degradation
and Pathways. J. Photochem. Photobiol. A Chem..

[48] Nason S. L., Lin E., Eitzer B., Koelmel J., Peccia J. (2022). Changes in Sewage Sludge
Chemical Signatures During a COVID-19 Community Lockdown, Part 1:
Traffic, Drugs, Mental Health, and Disinfectants. Environ. Toxicol. Chem..

[49] Saka C. (2022). Analytical
Methods on Determination in Pharmaceuticals and Biological Materials
of Chloroquine as Available for the Treatment of COVID-19. Crit. Rev. Anal. Chem..

[50] Fakayode S. O., Lisse C., Medawala W., Brady P. N., Bwambok D. K., Anum D., Alonge T., Taylor M. E., Baker G. A., Mehari T. F., Rodriguez J. D., Elzey B., Siraj N., Macchi S., Le T., Forson M., Bashiru M., Fernand Narcisse V. E., Grant C. (2024). Fluorescent Chemical Sensors: Applications
in Analytical, Environmental, Forensic, Pharmaceutical, Biological,
and Biomedical Sample Measurement, and Clinical Diagnosis. Appl. Spectrosc. Rev..

[51] He Q., Wang B., Liang J., Liu J., Liang B., Li G., Long Y., Zhang G., Liu H. (2023). Research on the Construction
of Portable Electrochemical Sensors for Environmental Compounds Quality
Monitoring. Mater. Today Adv..

[52] Maniglia B. C., Tapia-Blácido D. R. (2016). Isolation
and Characterization of
Starch from Babassu Mesocarp. Food Hydrocolloids.

[53] Zhao X., Li D., Wang L., Wang Y. (2023). Role of Gelation Temperature in Rheological
Behavior and Microstructure of High Elastic Starch-Based Emulsion-Filled
Gel. Food Hydrocolloids.

[54] Silva T. A., Moraes F. C., Janegitz B. C., Fatibello-Filho O. (2017). Electrochemical
Biosensors Based on Nanostructured Carbon Black: A Review. J. Nanomater..

[55] Tamanini F., Moraes B. S., Amaral C. S. T., Carvalho A. J. F., Trovatti E. (2023). Starch-Based
Orodispersible Film for Diclofenac Release. Braz. J. Pharm. Sci..

[56] Pavia, D. L. ; Lampman, G. M. ; Kriz, G. S. ; Vyvyan, J. R. Introduction to Spectroscopy, Belmont, USA 2001, p 13.

[57] Jovičić M., Bera O., Stojanov S., Pavličević J., Govedarica D., Bobinac I., Hollo B. B. (2023). Effects of Recycled
Carbon Black Generated from Waste Rubber on the Curing Process and
Properties of New Natural Rubber Composites. Polym. Bull..

[58] Debnath N., Panwar V., Roy T., Saha M., Pal K. (2020). Improved Dispersion
of Carbon Black in ABS/PANI Blend through Its Acid Functionalization
and Addition of Nanoclay, Thereby Enhancing Mechanical and Thermal
Properties. Polym. Bull..

[59] Zhu L., Jing X., Song L., Liu B., Zhou Y., Xiang Y., Xia D. (2014). Solid-Phase Synthesis
and Catalytic
Sweetening Performance of Sulfonated Cobalt Phthalocyanine from Sulfonated
Phthalic Anhydride Mixture. New J. Chem..

[60] Panwar V., Kumar P., Ray S. S., Jain S. L. (2015). Organic Inorganic
Hybrid Cobalt Phthalocyanine/Polyaniline as Efficient Catalyst for
Aerobic Oxidation of Alcohols in Liquid Phase. Tetrahedron Lett..

[61] Olejnik P., Gniadek M., Echegoyen L., Plonska-Brzezinska M. E. (2018). Nanoforest:
Polyaniline Nanotubes Modified with Carbon Nano-Onions as a Nanocomposite
Material for Easy-to-Miniaturize High-Performance Solid-State Supercapacitors. Polymers.

[62] Lohumi S., Kim M. S., Qin J., Cho B.-K. (2019). Improving Sensitivity
in Raman Imaging for Thin Layered and Powdered Food Analysis Utilizing
a Reflection Mirror. Sensors.

[63] Saravanan M., Ganesan M., Ambalavanan S. (2014). An in Situ
Generated Carbon as Integrated
Conductive Additive for Hierarchical Negative Plate of Lead-Acid Battery. J. Power Sources.

[64] Jiang S., Chen Z., Chen X., Nguyen D., Mattei M., Goubert G., Van Duyne R. P. (2019). Investigation
of Cobalt Phthalocyanine
at the Solid/Liquid Interface by Electrochemical Tip-Enhanced Raman
Spectroscopy. J. Phys. Chem. C.

[65] Yang H., Guo N., Xi S., Wu Y., Yao B., He Q., Zhang C., Wang L. (2024). Potential-Driven Structural Distortion
in Cobalt Phthalocyanine for Electrocatalytic CO2/CO Reduction towards
Methanol. Nat. Commun..

[66] Su J., Musgrave C. B., Song Y., Huang L., Liu Y., Li G., Xin Y., Xiong P., Li M. M.-J., Wu H., Zhu M., Chen H. M., Zhang J., Shen H., Tang B. Z., Robert M., Goddard W. A., Ye R. (2023). Strain Enhances the
Activity of Molecular Electrocatalysts via Carbon Nanotube Supports. Nat. Catal.

[67] Bauer B. A., Knorr D. (2004). Electrical Conductivity: A New Tool
for the Determination of High
Hydrostatic Pressure-Induced Starch Gelatinisation. Innovative Food Sci. Emerging Technol..

[68] Elgrishi N., Rountree K. J., McCarthy B. D., Rountree E. S., Eisenhart T. T., Dempsey J. L. (2018). A Practical Beginner’s
Guide to Cyclic Voltammetry. J. Chem. Educ..

[69] Bard Allen, J. ; Faulkner Larry, R. Electrochemical Methods: Fundamentals and Applications.; Wiley: New York, 2001.

[70] Zhang X.-F., Shao X. (2014). π–π
Binding Ability of Different Carbon Nano-Materials
with Aromatic Phthalocyanine Molecules: Comparison between Graphene,
Graphene Oxide and Carbon Nanotubes. J. Photochem.
Photobiol., A.

[71] Raeisi Z., Shayani-Jam H., Yaftian M. R., Farajmand B. (2025). Electrochemical
Oxidation of Hydroxychloroquine; Mechanistic Insights and Degradation
Pathways. J. Mol. Struct..

[72] Panić V., Stevanović R. M., Jovanović V. M., Dekanski A. B. (2008). Electrochemical
and Capacitive Properties of Thin-Layer Carbon Black Electrodes. J. Power Sources.

[73] Foster C., Pillay J., Metters J., Banks C. (2014). Cobalt Phthalocyanine
Modified Electrodes Utilised in Electroanalysis: Nano-Structured Modified
Electrodes vs. Bulk Modified Screen-Printed Electrodes. Sensors.

[74] Prabhu
C P K., Aralekallu S., Koodlur Sannegowda L. (2024). Efficacy of Phthalocyanine-Based
Catalysts in Electrochemical Sensors: A Comprehensive Review. Adv. Sens. Res..

[75] Schroeder R. L., Gerber J. P. (2014). Chloroquine and
Hydroxychloroquine Binding to Melanin:
Some Possible Consequences for Pathologies. Toxicol. Rep..

[76] Liberato P., Silva A., Okumura L., Diniz J., Oliveira A. (2020). Influência
da força iônica na estimativa de pK_a_ por
método voltamétrico. Quim. Nova.

[77] Gaolatlhe L., Barik R., Ray S. C., Ozoemena K. I. (2020). Voltammetric Responses
of Porous Co3O4 Spinels Supported on MOF-Derived Carbons: Effects
of Porous Volume on Dopamine Diffusion Processes. J. Electroanal. Chem..

[78] Monsef R., Salavati-Niasari M. (2022). Electrochemical
Sensor Based on a Chitosan-Molybdenum
Vanadate Nanocomposite for Detection of Hydroxychloroquine in Biological
Samples. J. Colloid Interface Sci..

[79] Mater
Mahnashi H., Mahmoud A. M., Saad Alkahtani A., El-Wekil M. M. (2021). Simultaneous Electrochemical Detection of Azithromycin
and Hydroxychloroquine Based on VS2 QDs Embedded N, S @graphene Aerogel/CCNTs
3D Nanostructure. Microchem. J..

[80] Zoubir J., Bakas I., Qourzal S., Tamimi M., Assabbane A. (2023). Electrochemical
Sensor Based on a ZnO-Doped Graphitized Carbon for the Electrocatalytic
Detection of the Antibiotic Hydroxychloroquine. Application: Tap Water
and Human Urine. J. Appl. Electrochem..

[81] Jeromiyas N., Mani V., Chang P.-C., Huang C.-H., Salama K. N., Huang S.-T. (2021). Anti-Poisoning Electrode for Real-Time in-Situ Monitoring
of Hydrogen Sulfide Release. Sens. Actuators,
B.

[82] Silva J. P. C., Santos-Neto D. R., Lopes C. E. C., Silva L. R. G., Dantas L. M. F., da
Silva I. S. (2025). A High Sensitivity Adsorptive-Electrochemical
Method for Rapid and Portable Determination of Hydroxychloroquine. J. Solid State Electrochem..

[83] Ferreira M. T., dos Santos Carvalho C. (2023). Chloroquine
and Hydroxychloroquine in the Environment
and Aquatic Organisms: A Review. Ambiente e
Agua—An Interdiscip. J. Appl. Sci..

[84] Mora A., Mahlknecht J., Rosales-Lagarde L., Hernández-Antonio A. (2017). Assessment
of Major Ions and Trace Elements in Groundwater Supplied to the Monterrey
Metropolitan Area, Nuevo León, Mexico. Environ. Monit Assess.

[85] Yavuz V. S., Kartal V., Sambito M. (2024). Comparative
Analysis of Water Quality
in Major Rivers of Türkiye Using Hydrochemical and Pollution
Indices. Water.

[86] Jarvie H. P., Neal C., Leach D. V., Ryland G. P., House W. A., Robson A. J. (1997). Major Ion Concentrations
and the Inorganic Carbon Chemistry
of the Humber Rivers. Sci. Total Environ..

[87] Marzouk-Trifi I., Baklouti L., Dammak L. (2023). Investigation of Calcium and Magnesium
Removal by Donnan Dialysis According to the Doehlert Design for Softening
Different Water Types. Membranes.

[88] Siqueira G. P., Araújo D. A. G., de Faria L. V., Ramos D. L. O., Matias T. A., Richter E. M., Paixão T. R. L.
C., Muñoz R. A. A. (2023). A Novel
3D-Printed Graphite/Polylactic Acid Sensor for the Electrochemical
Determination of 2,4,6-Trinitrotoluene Residues in Environmental Waters. Chemosphere.

