# Supplementary material for: Electrochemical Sensor Based on a Composite of Babassu Starch, Carbon Black, and Cobalt Phthalocyanine for the Detection of Hydroxychloroquine
Source: ACS Omega. 2026 Feb 12;11(7):12754–65. doi: 10.1021/acsomega.5c13354 (PMC12947185; doi:10.1021/acsomega.5c13354)
Supplement: Supplementary file 1 [file ao5c13354_si_001.pdf]

Supporting Information

Electrochemical sensor based on a composite of Babassu starch, carbon black, and cobalt phthalocyanine for the detection of hydroxychloroquine

João Pedro C. Silva[1,2], Gilvana P. Siqueira[2], Domingos Rosa Santos-Neto[1], Raquel G. Rocha[2], Jéssica Santos Stefano[1], Luiza Maria Ferreira Dantas[1], Luiz Ricardo G. Silva[1], Eduardo M. Richter [2], Rodrigo A. A. Muñoz[2*], Iranaldo S. da Silva [1**]

[1] *Department of Chemical Technology, Federal University of Maranhão, São Luís, Maranhão, 65080-805, Brazil.*

[2] *Institute of Chemistry, Federal University of Uberlândia, Uberlândia, Minas Gerais 38408-100, Brazil*

***Corresponding* authors:**

* munoz@ufu.br - ORCID: 0000-0001-8230-5825

** iranaldo.ss@ufma.br - ORCID: 0000-0001-6216-9141

Supporting Information

Figure S1A shows that the GCE naturally contains carbon atoms. The presence of oxygen atoms is also visible, which can be attributed to functional groups such as carboxyl and carbonyl that result from the oxidation of the glassy carbon surface layer during redox processes.[1] **Figure S1B** shows the elements Ca, Fe, Na, Al, K, Cl, O, and C, with a higher concentration of C compared to bare GCE, suggesting the presence of glucose structures from the starch film. The other elements are related to fibers, proteins, lipids, and minerals found in the mesocarp of the babassu coconut that were not eliminated during the extraction process.[2] **Figure S1C**, which depicts the surface of the CBSP/Starch/GCE, shows a higher intensity of carbon atoms than **Figures S1A and S1B** due to the presence of the CBSP nanomaterial on the electrode surface. **Figure S1D** shows the presence of the Co element, which was absent in the other images, indicating that the Co comes from the cobalt phthalocyanine in the modifier.

Supporting Information

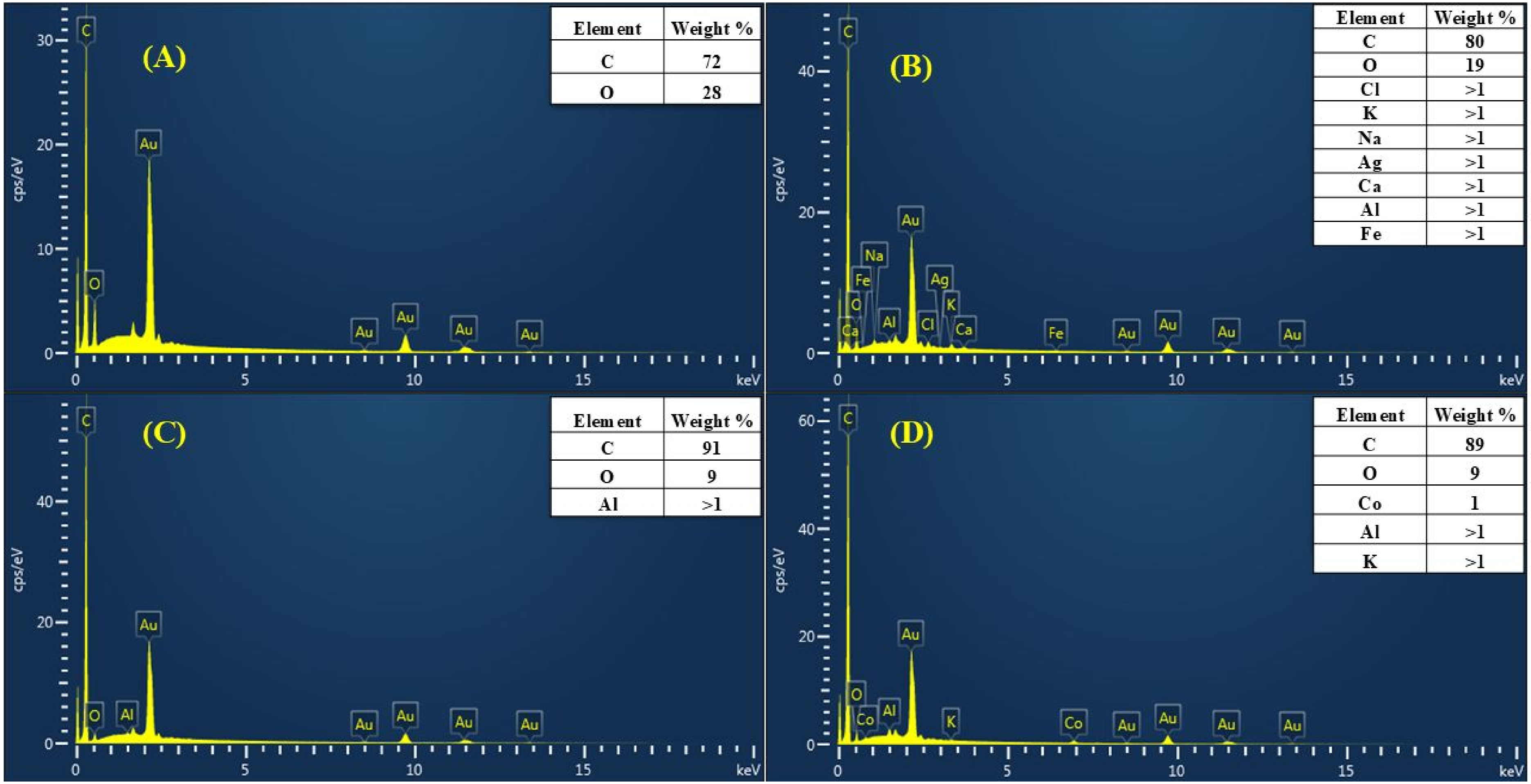

Figure S1. Energy Dispersive Spectroscopy (EDS) images of **(A)** GCE, **(B)** Starch/GCE, **(C)** CBSP/Starch/GCE, and **(D)** CoPC/CBSP/Starch/GCE.

Supporting Information

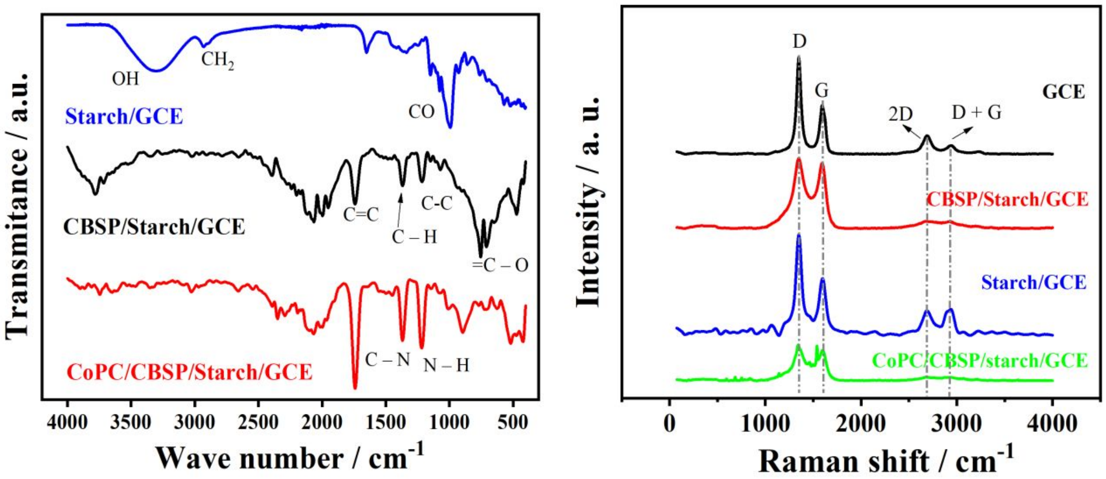

Figure S2. (A) FTIR spectra of Starch/GCE (blue), CBSP/Starch/GCE (black), and CoPc/CBSP/Starch/GCE (red); and **(B)** Raman spectra of GCE (black), Starch/GCE (blue), CBSP/Starch/GCE (red), and CoPc/CBSP/Starch/GCE (green).

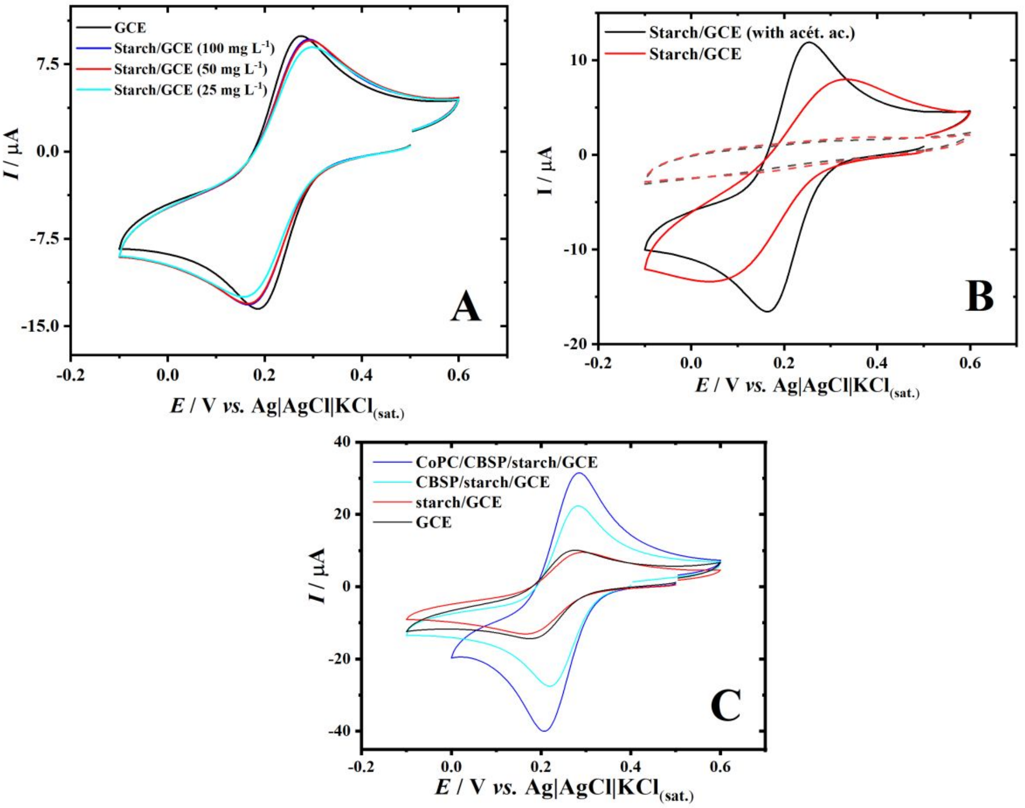

Figure S3. (A) CVs recorded in the presence of 1.0 mmol L^{-1} $[Fe(CN)_6]^{3-}$ in 0.5 mol L^{-1} KCl on GCE and Starch/GCE at concentrations of 25-100 mg L^{-1}. **(B)** CVs recorded in the presence of 1.0 mmol L^{-1} $[Fe(CN)_6]^{3-}$ in 0.5 mol L^{-1} KCl on Starch/GCE with acetic acid (black line) and without acetic acid (red line). **(C)** CVs recorded in 1.0 mmol L^{-1}

$[Fe(CN)_6]^{3-}$ in 0.1 mol L^{-1} KCl on GCE (black line), Starch/GCE (red), CBSP/Starch/GCE (indigo line), and CBSP/Starch/GCE/CoPc (blue line). Scan rate = 50 mV s^{-1}; E_{step} = 5 mV.

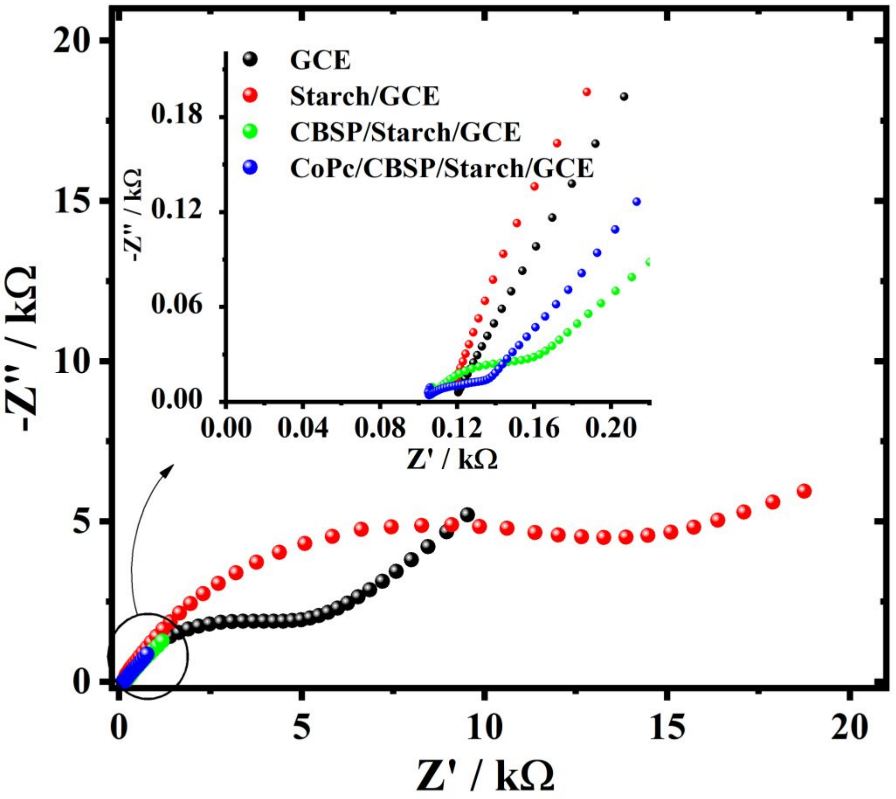

Figure S4. EIS spectra for bare GCE (●), (●) Starch/GCE, (●) CBSP/Starch/GCE, and (●) CoPc/CBSP/Starch/GCE.

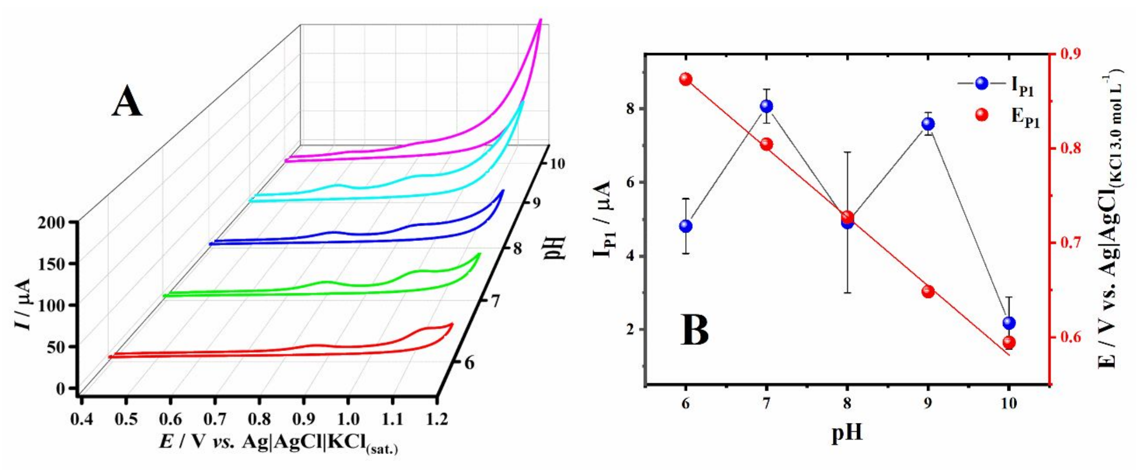

Figure S5. (A) CVs of CBSP/Starch/GCE/CoPc in the presence of HCQ (10 µmol L^{-1}) in BR buffer at varying pH values (pH 6.0-10.0). Scan rate = 50 mV s^{-1}; E_{step} = 5 mV; T_{PC} = 120 s. **(B)** Dependence of the peak current (I) (●) and peak potential (Ep) with the pH of the medium (●).

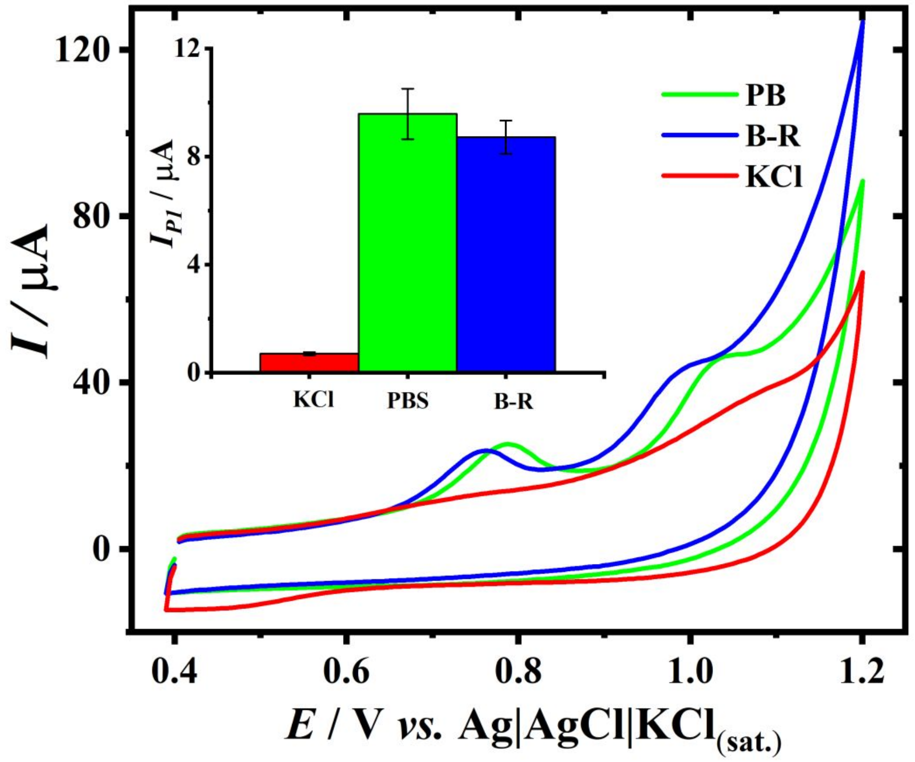

Figure S6. (A) CVs recorded at CBSP/Starch/GCE/CoPc in the presence of HCQ (10 µmol L^{-1}) using different supporting electrolytes. Scan rate = 50 mV s^{-1}; E_{step} = 5 mV; T_{PC} = 120 s.

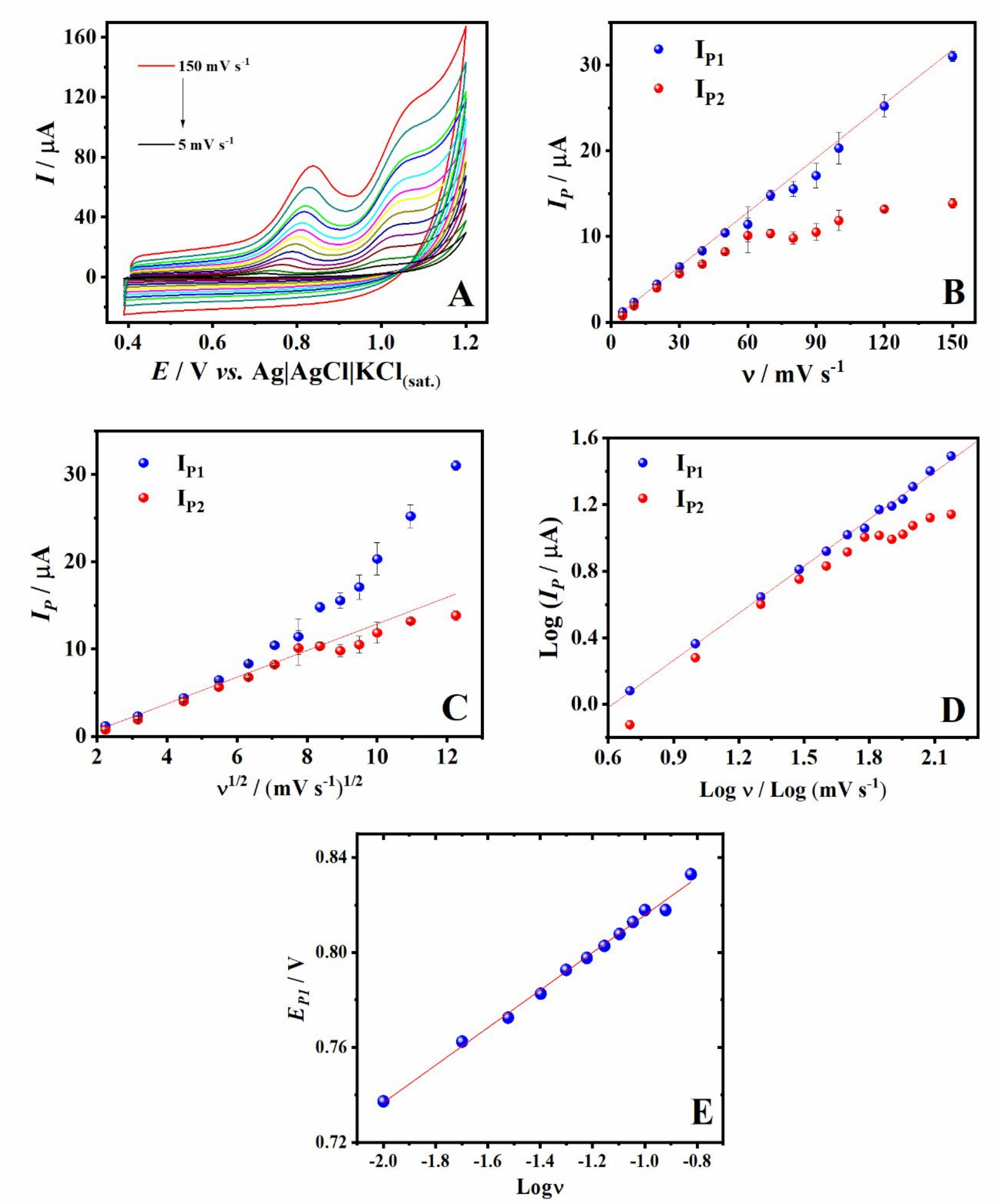

Figure S7. (A) CVs recorded on CBSP/Starch/CoPc/GCE with varying the scan rate (5-150 mV s^{-1}) in 0.1 mol L^{-1} PB (pH 7.0) in the presence of HCQ (10 µmol L^{-1}). Estep = 5 mV; T_{PC} = 120 s. **(B)** Dependence of the peak current (I_P) with scan rate (mV s^{-1}), n=3. **(C)** Dependence of Ip with the square root of scan rate (mV s^{-1})$^{1/2}$, n = 3. **(D)** Dependence of log(I_P) with log of scan rate (mV s^{-1}), n = 3. **(E)** Dependence of E_{P1} and log *ν* (V s^{-1}), n = 3.

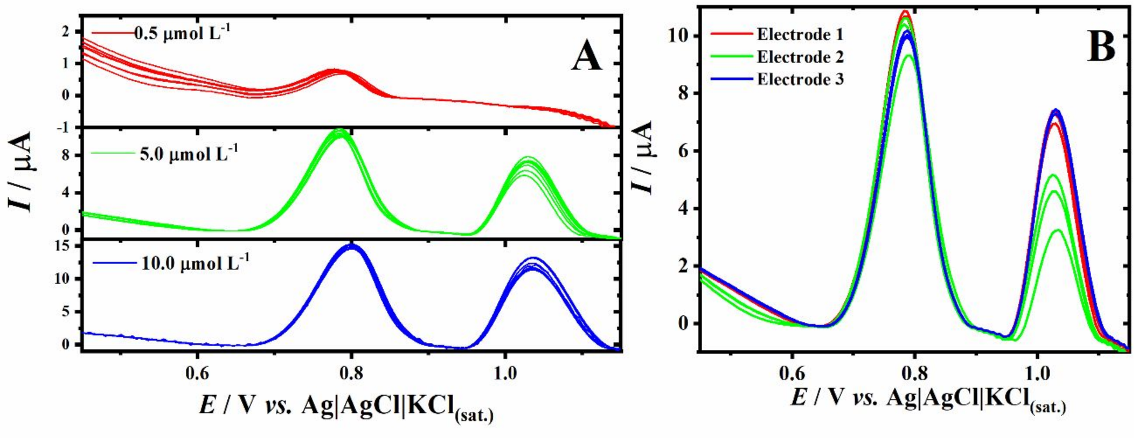

Figure S8. (A) LSV (baseline corrected) recorded in the presence of HCQ at concentrations of 0.5 μmol L^{-1}; 5.0 μmol L^{-1}; 10.0 μmol L^{-1}, using 0.1 mol L^{-1} PB (pH 7.0), as supporting electrolyte and CBSP/Starch/GCE/CoPc as the working electrode. (**B)** LSV (baseline corrected) recorded in the presence of in the presence of HCQ at a concentration of 5.0 μmol L^{-1} in 0.1 mol L^{-1} PB (pH 7.0), using three independently prepared CBSP/Starch/GCE/CoPc.

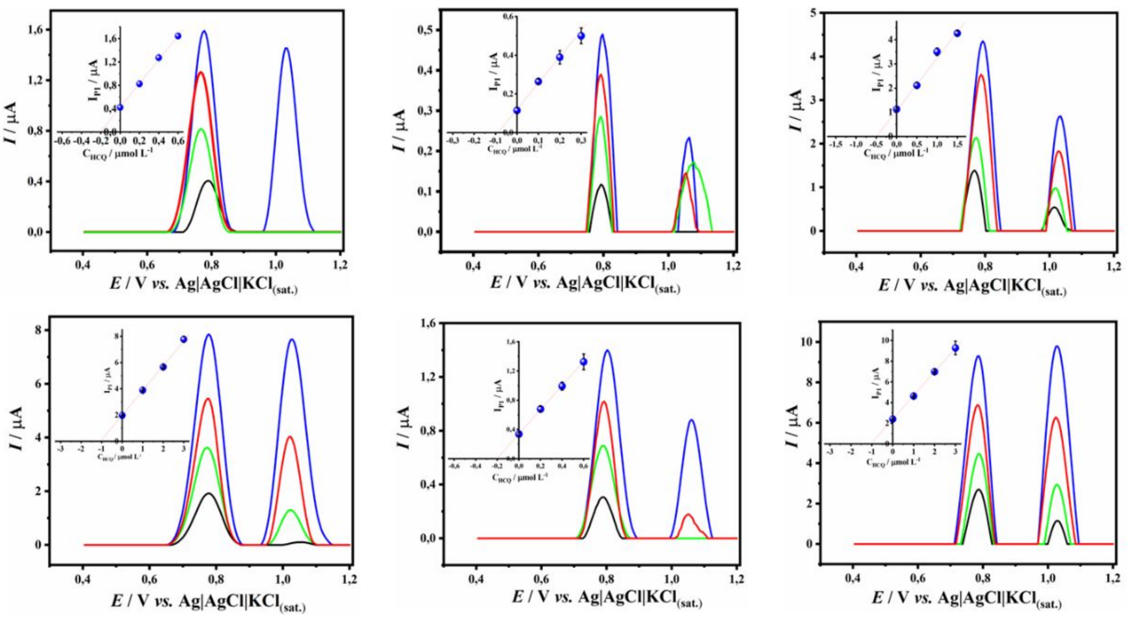

Figure S9. LSV (with baseline corrected) of CoPc/CBSP/Starch/GCE in phosphate buffer 0.1 mol L^{-1} (pH 7.0) in the presence of: **(A, B)** tap water; **(C, D)** water from a treatment plant, and **(E, F)** tablet sample with standard additions. ν = 100 mV s^{-1}; E_{step} = 5 mV; T_{PC} = 120 s.

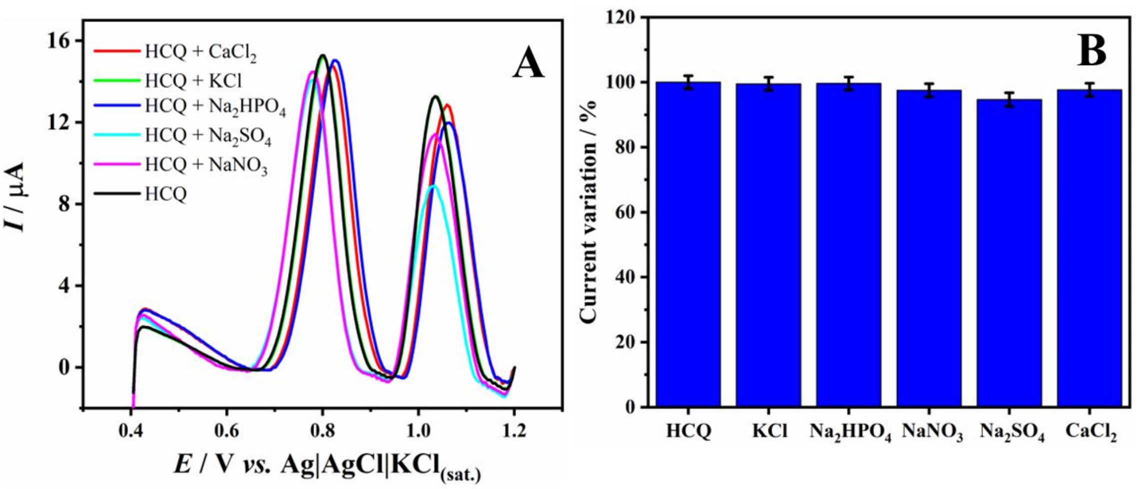

Figure S10. (A) LSV (baseline corrected) recorded in the presence of HCQ (10.0 μmol L^{-1}) using 0.1 mol L^{-1} PB (pH 7.0), as supporting electrolyte and CBSP/Starch/GCE/CoPc as the working electrode. Measurements were performed in the absence and in the presence of interfering salts, $CaCl_2$, KCl, Na_2HPO_4, Na_2SO_4, and $NaNO_3$, all added at a concentration of 10 mmol L^{-1}. ν = 100 mV s^{-1}; E_{step} = 5 mV; T_{PC} = 120 s. **(B)** Variation of HCQ peak current in the presence of interfering species, such as $CaCl_2$, KCl, Na_2HPO_4, $NaNO_3$ and Na_2SO_4, using HCQ/interfering agent ratio (1:1000).

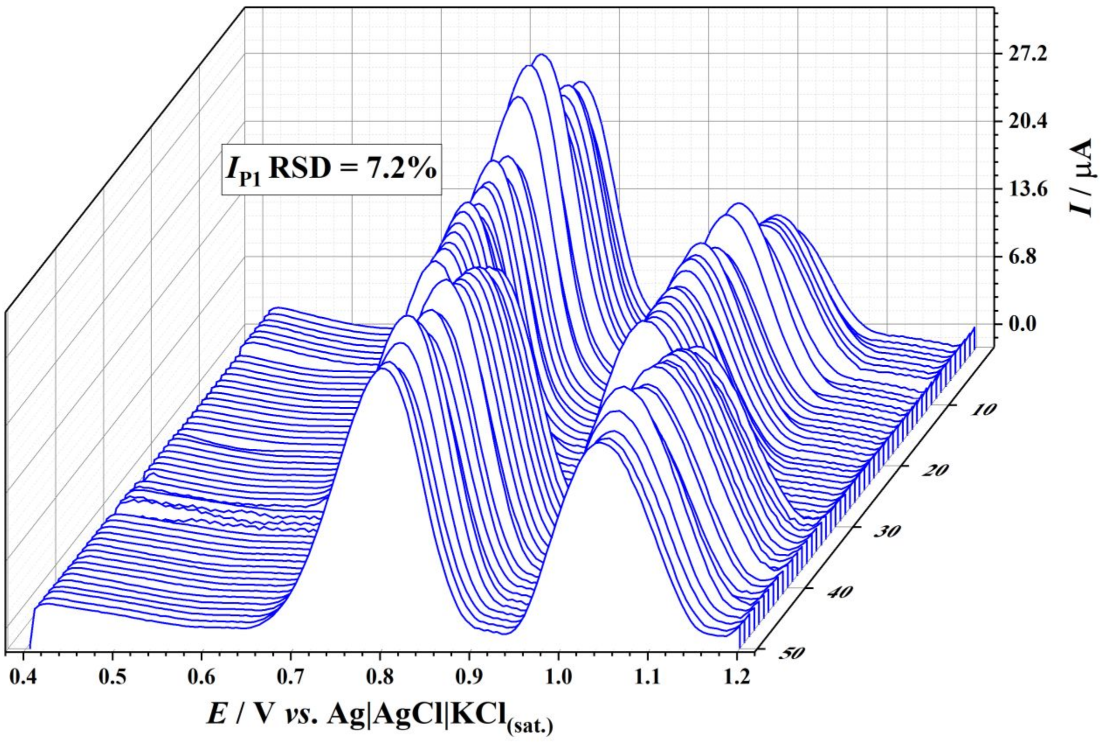

Figure S11. LSV (baseline corrected) recorded in the presence of HCQ at a concentration of 15.0 μmol L^{-1} in 0.1 mol L^{-1} PB (pH 7.0). ν = 100 mV s^{-1}; E_{step} = 5 mV; T_{PC} = 120 s.

Table S1. Comparison of ΔE_P and I_{PC} values between the different electrodes.

Electrode	ΔE_P / mV	I_{PC} / μA
CoPc/CBSP/Starch/GCE	65	−39.97
CBSP/Starch/GCE	65	−27.48
Starch/GCE	120	−13.06
Bare GCE	95	−14.29

Table S2. Comparison of the analytical characteristics of electrochemical sensors for HCQ detection.

Electrode	Technique	LOD (nmol L^{-1})	Linear Range (µmol L^{-1})	Ref.
MV-CHT/CPE[a]	DPV	0.22	0.0019 –194.0	3
VS 2 -QDs/GNA/CNT[b]	DPV	2.77	0.0084 – 0.22	4
GC-PMPDA[c]	LSV	4.65	0.09 – 10.21	5
ZnS/Ps@rGO/GCE[d]	DPV	0.46	0.005 – 0.065	6
BDD[e]	SWV	60.0	0.1 – 1.9	7
GC-PMPD SAM[f]	DPV	4.51	0.05 – 12.28	8
SPCB/GCE[g]	LSV	9.0	0.10 – 10.0	9
AuNPs@Cu-MOF/MWCNTs[h]	DPV	19.0	0.05 – 50.0	10
rGO-NPh@CPE[i]	DPV	50.0	0.1 – 1.0	11
PtNPs-MWCNTs/CPE[j]	SWV	28.0	0.099–7.1	12
ZnO-NprGO/CPE[k]	SWV	57.0	0.07 – 5.5	13
CoPc/CBSP/Starch/GCE	LSV	15.0	0.10 – 16.0	This paper

a Carbon paste electrode modified with $V_{3.6}Mo_{2.4}O_{16}$-chitosan nanocomposite; **b** Vanadium disulfide quantum dots and insertion into 3D N. S-doped graphene aerogel and carbon nanotube nanostructure; **c** Glassy carbon electrode modified with a self-assembled monolayer of N.N′-bis[(E)-(1-pyridyl)methylidene]-1.3-propanediamine; **d** Glassy carbon electrode modified with zinc sulfide nanoparticles and reduced graphene oxide; **e** Boron-doped diamond electrode; **f** Self-assembled monolayer of N.N′-bis[(E)−(1−pyridyl)methylidene]−1.3−propanediamine on an unmodified glassy carbon electrode; **g** Glassy carbon electrode modified with Carbon Black Super P. **h** Screen-printed carbon electrode (SPCE) modified with a hierarchical nanocomposite of gold

nanoparticles (AuNPs), copper-based metal–organic frameworks (Cu-MOFs), and multi-walled carbon nanotubes (MWCNTs); **i** carbon paste electrode (CPE) modified with a composite of natural phosphate (NPh) and reduced graphene oxide; **j** carbon paste electrode (CPE) modified with multi-walled carbon nanotubes (MWCNTs) and platinum nanoparticles (Pt NPs); **k** Zinc oxide nanoparticles decorated nitrogen doped porous reduced graphene oxide-based hybrid.

REFERENCES

(1) Dekanski, A.; Stevanović, J.; Stevanović, R.; Nikolić, B. Ž.; Jovanović, V. M. Glassy Carbon Electrodes. *Carbon N Y* **2001**, *39* (8), 1195–1205. https://doi.org/10.1016/S0008-6223(00)00228-1.

(2) Borges, L. A.; Ramos, K. K.; Felisberto, M. H. F.; Franciosi, E. R. N.; Efraim, P. Babassu Mesocarp: A Sustainable Source for Obtaining Starch and New Products. *Starch - Stärke* **2023**, *75* (7–8). https://doi.org/10.1002/star.202200203.

(3) Monsef, R.; Salavati-Niasari, M. Electrochemical Sensor Based on a Chitosan-Molybdenum Vanadate Nanocomposite for Detection of Hydroxychloroquine in Biological Samples. *J Colloid Interface Sci* **2022**, *613*, 1–14. https://doi.org/10.1016/J.JCIS.2022.01.039.

(4) Mater Mahnashi, H.; Mahmoud, A. M.; Saad Alkahtani, A.; El-Wekil, M. M. Simultaneous Electrochemical Detection of Azithromycin and Hydroxychloroquine Based on VS2 QDs Embedded N, S @graphene Aerogel/CCNTs 3D Nanostructure. *Microchemical Journal* **2021**, *163*, 105925. https://doi.org/10.1016/J.MICROC.2021.105925.

(5) Khoobi, A.; Ghoreishi, S. M.; Behpour, M.; Shaterian, M.; Salavati-Niasari, M. Design and Evaluation of a Highly Sensitive Nanostructure-Based Surface Modification of Glassy Carbon Electrode for Electrochemical Studies of Hydroxychloroquine in the Presence of Acetaminophen. *Colloids Surf B Biointerfaces* **2014**, *123*, 648–656. https://doi.org/10.1016/J.COLSURFB.2014.10.002.

(6) Alkahtani, S. A.; Mahmoud, A. M.; Mahnashi, M. H.; AlQarni, A. O.; Alqahtani, Y. S. A.; El-Wekil, M. M. Facile One Pot Sonochemical Synthesis of Layered Nanostructure of ZnS NPs/RGO Nanosheets for Simultaneous Analysis of Daclatasvir and Hydroxychloroquine. *Microchemical Journal* **2021**, *164*, 105972. https://doi.org/10.1016/J.MICROC.2021.105972.

(7) Deroco, P. B.; Vicentini, F. C.; Oliveira, G. G.; Rocha-Filho, R. C.; Fatibello-Filho, O. Square-Wave Voltammetric Determination of Hydroxychloroquine in Pharmaceutical and Synthetic Urine Samples Using a Cathodically Pretreated Boron-Doped Diamond Electrode. *Journal of Electroanalytical Chemistry* **2014**, *719*, 19–23. https://doi.org/10.1016/J.JELECHEM.2014.01.037.

(8) Ghoreishi, S. M.; Behpour, M.; Khoobi, A.; Salavati-Niasari, M. Electrochemical Study of a Self-Assembled Monolayer of N,N′-Bis[(E)-(1-Pyridyl) Methylidene]-1,3-Propanediamine Formed on Glassy Carbon Electrode: Preparation, Characterization and Application. *Analytical Methods* **2013**, *5* (23), 6727–6733. https://doi.org/10.1039/C3AY41480A.

(9) Silva, J. P. C.; Santos-Neto, D. R.; Lopes, C. E. C.; Silva, L. R. G.; Dantas, L. M. F.; da Silva, I. S. A High Sensitivity Adsorptive-Electrochemical Method for Rapid and Portable Determination of Hydroxychloroquine. *Journal of Solid State Electrochemistry* **2024**. https://doi.org/10.1007/s10008-024-06032-z.

(10) Feng, X.; Zhao, J.; Wu, S.; Kan, Y.; Li, H.; Zhang, W. An Electrochemical Sensor Based on AuNPs@Cu-MOF/MWCNTs Integrated Microfluidic Device for Selective Monitoring of Hydroxychloroquine in Human Serum. *Chemosensors* **2025**, *13* (6), 200. https://doi.org/10.3390/chemosensors13060200.

(11) Chhaibi, B.; Matrouf, M.; Elhaddioui, L.; Berd, A.; Laghrib, F.; Saqrane, S.; Bakasse, M.; Lahrich, S.; Farahi, A.; EL Mhammedi, M. A. Thermal Synthesis of Reduced Graphene Oxide-Natural Phosphate for Hydroxychloroquine Detection in Urine, Wastewater, and Pharmaceutical Samples. *J Electrochem Soc* **2025**, *172* (6), 067501. https://doi.org/10.1149/1945-7111/addc43.

(12) Feitosa, M. H. A.; Santos, A. M.; Wong, A.; Sotomayor, M. D. P. T.; Barros, W. R. P.; Lanza, M. R. V.; Moraes, F. C. Enhancing Hydroxychloroquine Detection Using Carbon Paste Electrode Modified with Platinum Nanoparticles and MWCNTs. *J Appl Electrochem* **2025**, *55* (8), 2265–2276. https://doi.org/10.1007/s10800-025-02293-2.

(13) Amiri, M.; Hashemi, Z.; Chekin, F. Zinc Oxide Nanoparticles Decorated Nitrogen Doped Porous Reduced Graphene Oxide-Based Hybrid to Sensitive Detection of Hydroxychloroquine in Plasma and Urine. *J Mater Sci Mater Med* **2025**, *36* (1), 4. https://doi.org/10.1007/s10856-024-06847-2.
